# SIFD-associated TRNT1 deficiency unveils importance of TSPO during macrophage antibacterial and antiviral responses

**DOI:** 10.3389/fimmu.2025.1497766

**Published:** 2025-09-11

**Authors:** Duale Ahmed, Angelo Slade, Thet Fatica, Stephen Baird, Krishna Bhattarai, Thérèse Atallah, Edana Cassol, Martin Holcik

**Affiliations:** ^1^ Department of Health Sciences, Carleton University, Ottawa, ON, Canada; ^2^ Children’s Hospital of Eastern Ontario Research Institute, Ottawa, ON, Canada

**Keywords:** macrophage, SIFD, TRNT1, immunodeficiency, mitochondria, immune responses, TSPO

## Abstract

**Introduction:**

Mitochondria support cellular biosynthetic and bioenergetic demands and mediate cell signaling. Their dysfunction is implicated in a wide range of diseases, including congenital disorders. One such disorder, sideroblastic anemia with B-cell immunodeficiency, periodic fevers, and developmental delay (SIFD), is caused by mutations in the tRNA-nucleotidyltransferase enzyme TRNT1. While SIFD is known to affect immune function, the role of macrophages—key mediators between innate and adaptive immunity—remains underexplored.

**Methods:**

To investigate the impact of TRNT1 deficiency on macrophage function, we employed siRNA-mediated knockdown of TRNT1 in murine RAW264.7 macrophages. Cells were stimulated with lipopolysaccharide (LPS) and Polyinosinic:polycytidylic acid (Poly (I:C)) to mimic bacterial and viral infections, respectively. Cytokine production was measured, and mitochondrial reprogramming was assessed. Bioinformatic analysis was conducted to identify TRNT1-dependent transcripts, focusing on mitochondrial-associated proteins. Functional rescue experiments were performed using TSPO ligands and TSPO overexpression.

**Results:**

TRNT1 knockdown impaired inflammatory cytokine production in response to both LPS and Poly (I:C). This correlated with diminished mitochondrial reprogramming, suggesting a mechanistic link between TRNT1 activity and macrophage effector function. Transcriptomic analysis identified the mitochondrial translocator protein (TSPO) as a TRNT1-dependent gene. TSPO expression was differentially regulated following stimulation in TRNT1-deficient cells. While TSPO ligand activation failed to restore cytokine production, TSPO overexpression prior to TRNT1 knockdown selectively rescued the inflammatory response to Poly (I:C), but not LPS. This rescue was associated with enhanced recruitment of VDAC to the mitochondrial permeability transition pore via TSPO.

**Discussion:**

Our findings reveal that TRNT1 is critical for pathogen-specific mitochondrial reprogramming in macrophages, influencing their inflammatory capacity. The differential restoration of cytokine responses via TSPO overexpression underscores the complexity of mitochondrial signaling in immune regulation. These insights suggest that targeting mitochondrial pathways may offer a novel therapeutic strategy for managing immunodeficiency in SIFD.

## Introduction

Mitochondrial dysfunction is a pathological characteristic of a wide spectrum of disorders ranging from aging to most chronic diseases such as Alzheimer’s, Parkinson’s and atherosclerosis to name a few (1, 2). Some diseases associated with mitochondrial dysfunction are also genetic in nature, caused by specific mutations in genes within the mitochondrial and nuclear genomes that are involved in various cellular processes such as energy metabolism, nucleic acid metabolism, genetic instability, and protein processing and synthesis (3). These diseases typically manifest with a wide array of symptoms. While some can be directly attributed to the primary mitochondrial dysfunction, others present as secondary symptoms that may not initially appear connected to the original mutation (4). As such, mitochondrial diseases are extremely complicated disorders to understand, thus requiring further investigation and resources to dissect and examine the links between the genetic mutations and the corresponding mitochondrial dysfunction.

The syndrome of congenital sideroblastic anemia, B-cell immunodeficiency, periodic fevers, and developmental delay (SIFD) is a relatively new mitochondrial disease linked to mutations within the gene of the tRNA-maturating enzyme TRNT1, which catalyzes the addition and the maintenance of the conserved nucleotide triplet CCA to the 3’ terminus of all tRNAs which allows for the incorporation of the proper amino acid onto its respective tRNA (4–9). In addition, TRNT1 serves multiple “moon-lighting” roles in cells from preventing the use of damaged tRNAs during translation, assessing the structural integrity of tRNAs, and tRNA nuclear transport (10–12). Those suffering from SIFD are immunologically characterized by recurrent inflammatory episodes due to autoinflammatory responses and significant deficiencies in mature B cells and serum immunoglobulin (Ig) levels (5, 13–15). Yet, little work has been done investigating the effects of SIFD and TRNT1-associated mutations on other aspects of the immune system. In patient fibroblasts, we showed that they have reduced assembly of the electron transport chain (ETC) complexes, which likely contributes to the mitochondrial defects and reduced oxidative phosphorylation (13). These cells are also sensitive to oxidative stress, linked to elevated angiogenin-dependent cleavage of tRNAs (16). Giannelou and colleagues (15) found that while patient fibroblasts possess a significant deficiency in mature tRNAs, the associated mitochondrial defects result in increased production of mitochondrial reactive oxygen species (mtROS) in patient fibroblasts. Furthermore, they identified an inability to upregulate protein clearance pathways in TRNT1-deficient cells which is linked to the autoinflammatory release of IL-6 and IFN-γ into plasma (15). While the link between TRNT1 deficiency and mitochondrial dysfunction is clearly established (13, 15), its connections to SIFD-mediated immune dysfunction are more elusive and require further study.

Macrophages are dynamic and heterogeneous cells found in almost every tissue of the body. They perform a multitude of roles during an immune response: surveying and recognizing pathogens using pathogen recognition receptors (PRRs) to mount an immune response using cellular processes such as phagocytosis, cytokine production, antigen presentation, adaptive immune cell activation as well as re-establishing homeostasis by promoting tissue remodeling and repair (17–21). Critical to these diverse macrophage functions is the dynamic modulation of mitochondria. In addition to its role in supporting the bioenergetic and biosynthetic demands of the cell, mitochondria are vital for innate sensing and function by acting as a scaffold for signaling events and producing bioactive molecules that have direct and indirect effects on innate immune functions (22–27). These mitochondrial functions allow macrophages to deliver tightly regulated pathogen-specific responses and limit excess damage due to inflammation (28). Yet, their functions in mitochondrial diseases such as SIFD have been poorly understood.

Here, we evaluated how TRNT1 deficiency affects macrophage function. We discovered that when we used siRNA to silence TRNT1 in a macrophage cell line, there was a notable reduction in its ability to mount an inflammatory response in cells stimulated with either Lipopolysaccharide (LPS) or Polyinosinic:polycytidylic acid (Poly (I:C) or PIC), two widely recognized TLR ligands that mimic bacterial and viral responses, respectively. This reduction is closely associated with a decrease in the cell’s ability to reprogram its mitochondria, with decreased respiration, mitochondrial membrane potential (MMP), mitochondrial abundance, mitochondrial permeability transition pore (mPTP) activity, and altered ETC expression. Bioinformatic analysis of mitochondrial-related mRNA transcripts identified the mitochondrial translocator protein (TSPO) as a candidate protein most affected by the loss of TRNT1. Upon further analysis, TSPO was found to have differential effects during LPS and Poly (I:C) engagement, where TSPO expression is maintained in Poly (I:C)-stimulated but not LPS-stimulated siTRNT1 cells. Stabilizing the protein structure with the TSPO ligand PK11195 recovers TSPO expression in LPS-stimulated siTRNT1 cells and sustains respiration in both LPS and Poly (I:C) treated siTRNT1 cells but was not sufficient to restore the inflammatory response. However, overexpression of TSPO before TRNT1 knockdown can recover macrophage effector responses only in Poly (I:C)-treated siTRNT1 cells, which is linked to increased recruitment of the Voltage-dependent anion channel (VDAC) into the mPTP. This suggests that the mitochondrial reprogramming required for specific pathogenic responses is impaired in SIFD patients but may be recoverable. These findings suggest that TSPO is a potential therapeutic target for augmenting specific immune responses for those diagnosed with SIFD.

## Materials and methods

### Reagents

LPS (Cat#: TLRL-EKLPS) and high molecular weight Poly (I:C) (Cat#: TLRL-PIC) were purchased from InvivoGen. Antimycin A (AA) (Cat#: A8674), rotenone (ROT) (Cat#: R8875), oligomycin (Oligo) (Cat#: O4876) and carbonyl cyanide-p-trifluoromethoxyphenylhydrazone (FCCP) (Cat#: C2920) were acquired from Sigma-Aldrich. IL-1β (Cat#: DY401-05), IL-6 (Cat#: DY406-05), TNF (Cat#: DY410-05), IFN-β (Cat#: DY8234-05) DuoSet ELISA kits were purchased from R&D Systems. Tetramethylrhodamine, methyl ester (TMRM) (Cat#: I34361), Image-IT™ LIVE Mitochondrial Transition Pore Assay Kit (Cat#: I35103), MitoTracker™ Green (Cat#: M7514), MitoSOX™ Red (Cat#: M36008) and CellROX™ Orange (Cat#: C10443) probes were from ThermoFisher. Antibodies against core proteins of Complexes I-V (NDUFB8, SDHB, UQCRC2, COXIV (Cat#: A21348), ATP5A (Part of an antibody cocktail with Complexes I-III; Cat#: 458099)) and TSPO (Cat#: MA5-24844) were received from ThermoFisher. Antibodies targeting SOD2 (Cat#: 13194S), GPX4 (Cat#: 52455S), UCP2 (Cat#: 89326S), and VDAC (Cat#: 4661S) were acquired from Cell Signaling Technology. Anti-TRNT1 (Cat#: NBP1-86589) was bought from Novus Biologicals. All Stars Negative Control siRNA (Cat#: 1027281) and FlexiTube GeneSolution TRNT1-specific siRNA (Cat#: GS70047) were obtained from Qiagen. PK11195 (Cat#: 10191-264) was purchased from BioVision. A plasmid encoding mouse TSPO coding sequence (Cat#: MC207241) and an empty mammalian plasmid (PS100005) were received from OriGene.

### Animals and macrophage cell culture

All animal procedures were approved by the Carleton University Animal Care Committee and were conducted in accordance with the guidelines provided by the Canadian Council for Animal Care. The bone marrow cells from the tibias and femurs of 12-week-old C57BL/6 female mice were isolated and cryopreserved in a 90% FBS/10% DMSO solution until needed. As described previously (29), mouse bone marrow progenitors were differentiated over 10 days in Dulbecco’s modified Eagle’s medium (DMEM) (Gibco; Cat#: 11995-073) containing 15% L929 fibroblast cell-conditioned medium, 10% fetal bovine serum (FBS) (Gibco; Cat#: 10437-028) and 1% Penicillin-Streptomycin (PenStrep) (Gibco; Cat#: 15140-122) on 100mm non-tissue culture coated Petri dishes. On day 10, the differentiated bone marrow-derived macrophages (BMDMs) left in a biosafety cabinet to sit for 1 hour before detachment with PBS. Cells were counted and seeded onto tissue culture or non-tissue culture treated plates at a concentration of 1 x 10^6^ cells/mL and allowed to rest overnight.

Mouse macrophage cell line RAW264.7 cells (Cat#: TIB-71), and Human monocyte/macrophage THP-1 cell line (Cat#: TIB-202) were obtained from the American Type Culture Collection (ATCC). RAW264.7 cells were cultured in DMEM containing 10% FBS and 1% PenStrep. THP-1 cells were cultured in Roswell Park Memorial Institute medium (RPMI 1640) (Cat#: 21870-076) supplemented with 10% FBS and 1% PenStrep. Cells were maintained in Nunc filter-capped flasks (ThermoFisher) until the start of experiments. To begin, RAW264.7 cells were scrapped, collected, counted, and plated onto tissue culture-coated or non-tissue culture coated 12-well plates, depending on the experiment. THP-1 cells were collected, counted and plated onto tissue culture-coated or non-tissue culture coated 12-well plates, depending on the experiment. Cell passage number did not exceed fifteen for all experiments.

### siRNA knockdown of TRNT1

A general diagram outlines the experimental workflow can be found in **Supplementary Figure 4**. THP-1 cells were plated onto 12-well tissue culture-coated plates at a density of 4x10^5^ cells per well and were either differentiated with 20ng/mL Phorbol 12-myristate 13-acetate (PMA) or left undifferentiated for 48 hrs. Afterwards, cells were transfected with either 20nM All Star Negative control siRNA (referred going forward as siCtrl or siC) or FlexiTube GeneSolution GS51095 siRNA, consisting of a cocktail of four siRNAs targeting human TRNT1 used at 5nM each (referred going forward as siTRNT1 or siT). RAW264.7 cells were seeded in DMEM at a density of 6x10^4^ cells per well onto 12-well tissue culture-coated plates. After seeding, cells were transfected with either 20nM All Star Negative control siRNA (siCtrl or siC) or FlexiTube GeneSolution GS70047 siRNA, which consists of a cocktail of four siRNAs targeting mouse TRNT1 used at 5nM each (siTRNT1 or siT). Mouse BMDMs were plated in 12-well tissue culture-coated plates at a density of 5x10^5^ cells per well and transfected using the same procedure as the RAW264.7 cells.

All transfections were conducted in Opti-MEM™ I Reduced-Serum Medium (Gibco; Cat#: 31985-070) without FBS or antibiotics and complexed with Lipofectamine RNAiMax (ThermoFisher; Cat#: 13778-075) before being added to cells cultured in DMEM onto a 12-well plate. 48 hours after transfection, cells were washed with phosphate buffered saline (PBS) (ThermoFisher; Cat#: 10010-049) before treatment with either 100ng/mL (LPS) or 10µg/mL Poly (I:C) for 2, 6, and 24 hrs. LPS and Poly (I:C) were selected to represent responses to bacterial and viral infections, respectively. In addition, both concentrations were selected to produce a robust inflammatory response (28) akin to inflammatory responses seen in SIFD patients.

To confirm the effects are due to the loss of TRNT1 protein, a mammalian expression vector pCMV6-AC-DDK with an inserted full ORF sequence of human TRNT1, previously described by our group (6) was used to rescue TRNT1 expression in RAW264.7 cells following knockdown. Cells were plated in DMEM at a density of 5x10^4^ cells per well onto 12-well tissue culture-coated plates. After a 48-hour transfection with siCtrl or siTRNT1, cells were transfected with 2µg of pCMV6-AC-DKK+TRNT1 (pTRNT1) vector complexed with Lipofectamine™ LTX with PLUS™ reagent (ThermoFisher; Cat#: 15338-030) in Opti-MEM. Afterwards, cells were treated with 100ng/mL LPS or 10µg/mL Poly (I:C) for 24 hours before further downstream analyses were done.

### Genome-wide analysis of TRNT1-affected transcripts

A human reference genome was downloaded in a fasta file format from the National Center for Biotechnology Information’s (NCBI) Human Genome Resources webpage (https://www.ncbi.nlm.nih.gov/genome/guide/human). Using the BioPerl module on a Linux OS, a Perl script was developed to search and determine the percentage of key codons of interest (ACG, GCA, TTA, CTT, CTA, CTG, and CTC) found in all mRNA transcripts within the human genome. In SIFD patient fibroblasts, tRNAs corresponding to these codons were found to be significantly reduced compared to healthy patient controls (16). The generated list of transcripts was filtered further to isolate all transcripts from mitochondrial-related genes using the MitoCarta3.0 database, which provided detailed information on the ~1100 genes identified by combining data from mass spectrometry of mitochondrial samples, large-scale GFP tagging/microscopy, and multiple genome-scale datasets of mitochondrial localization (30). All mitochondrial transcripts were sorted for those that possess the highest percentage of codons of interest within its sequence. Differentially affected transcripts were defined as having a percentage point 2 standard deviations higher than the mean percentage point of all transcripts (Mean = 12.603 ± 2.282) (**Supplementary Table 1**). Genes of interest were manually identified and selected for further analysis.

### Impact of TSPO ligand activation and TSPO overexpression on RAW264.7 function

TRNT1 knockdown was done as described above (see “siRNA knockdown of TRNT1” section). After knockdown, transfected RAW264.7 cells were co-treated with 10nM PK11195 and either 100ng/mL LPS or 10µg/mL Poly (I:C) for 24 hours before downstream analyses were conducted.

Mammalian expression vector pCMV6-Entry containing a full open reading frame sequence of mouse TSPO gene was used to overexpress TSPO in RAW264.7 cells. 5x10^4^ cells per well in DMEM were seeded onto 12-well tissue culture-coated plates. After seeding, cells were transfected with 0.6µg per well of pCMV6-Entry_TSPO (pTSPO) vector complexed with Lipofectamine™ LTX with PLUS™ reagent in Opti-MEM. After 24 hours, cells were transfected for 48 hours with either siCtrl or siTRNT1 as described above (see “siRNA knockdown of TRNT1” section). Afterwards, cells were treated with either 100ng/mL LPS or 10µg/mL Poly (I:C) for 24 hours before further downstream analyses were conducted.

### Quantification of cytokine production in culture supernatants via ELISA

Cell culture supernatants were collected following stimulation with either LPS or Poly (I:C) and spun at 14,800 RPM for 10 mins to remove cellular debris. IL-1β, IL-6, IL-10, IFN-β levels were measured *via* ELISA, according to the manufacturer’s instructions.

### Evaluation of mitochondrial function, ROS & cell viability via flow cytometry

RAW264.7 cells that were designated for flow cytometry analysis were plated onto 12-well non-tissue culture-coated plates at the same density and treated as above (see “siRNA knockdown of TRNT1” or “Impact of TSPO ligand activation and TSPO overexpression on RAW264.7 function” sections). After being treated with LPS or Poly (I:C), cells were left at room temperature in a biosafety cabinet for 1 hour before being detached with PBS and subsequently stained with fluorescent probes according to manufacturer’s instructions. Differences in MMP were evaluated using 10nM TMRM in complete DMEM. Changes in mitochondrial abundance were measured using 150nM MitoTracker™ Green in DMEM without FBS. Mitochondrial and cellular ROS were monitored using 2.5μM MitoSOX™ Red in PBS and 5μM CellROX™ Orange in complete DMEM, respectively. Mitochondrial permeability transition pore (mPTP) activity was measured using the Image-IT™ LIVE Mitochondrial Transition Pore Assay Kit (160nM Calcein AM diluted in Hank’s Balanced Salt Solution (HBSS) w/Ca^2+^ [ThermoFisher; Cat#: 14025-092]). Regardless of the probe used, all cells were incubated at 37°C for 30 mins prior to washing and subsequent single-cell analysis using an Attune NxT Flow Cytometer (ThermoFisher). A minimum of 10,000 events per sample were captured by the flow cytometer, and the data collected was analyzed using FlowJo Software (version 10.0.7). After gating upon the RAW264.7 cell population based on size (*via* forward scatter (FSC) and side scatter (SSC)), then gating upon the single cell population (*via* SSC-H vs. SSC-A scatter plot), events were characterized based on the respective fluorescent profiles of each probe. In experiments using TMRM, MitoSOX Red and MitoTracker™ Green, subpopulations were categorized as either “positive” cells (high fluorescence) or “negative” cells (low fluorescence) for their respective probe. TMRM and MitoTracker™ Green data were presented as the median fluorescence intensity (MFI) of the “positive” population. TMRM data was also presented as the percentage of cells with non-active mitochondria using the “TMRM negative” population. MitoSOX™ Red data were presented as the percentage of “MitoSOX™ Red positive” cells. For experiments in which the Mitochondrial Transition Pore Assay, CellROX™ Orange and MitoTracker™ Green probes were used, samples were characterized based on changes in the MFI of the total population. The MFI value of each sample was normalized to its respective control sample and presented as a fold change (FC) value. An example of the gating strategies mentioned above can be found in **Supplementary Figure 5**.

To verify that the TRNT1 knockdown does not result in loss in cell viability, treated RAW264.7 cells were detached from 12-well non-tissue culture-coated plates with PBS and resuspended to a concentration of 1x10^6^ cells/mL with PBS. Cells were stained with 4µg/mL Propidium Iodide (PI) (ThermoFisher; Cat#: P1304MP), mixed gently, and placed on ice for 15 mins before single-cell analysis was done using an Attune NxT Flow Cytometer. A minimum of 10,000 events per sample were captured by the flow cytometer, and the data collected was analyzed using FlowJo Software. The initial gating strategy follows what was described in the previous paragraph up to the identification of the single cell population. Subpopulations were categorized as either “PI positive” cells or “PI negative” cells. PI data was presented as the percentage of “PI positive” cells.

### Evaluation of protein expression via western blot analysis

Following transfection/treatment of cells, denatured protein lysates were collected using Pierce’s radioimmunoprecipitation assay (RIPA) (ThermoFisher; Cat#: 89900) buffer supplemented with Halt™ Protease and Phosphatase Inhibitor Cocktail (ThermoFisher; Cat#: 78441). The total protein concentration in each sample was determined using the DC Assay (Bio-Rad Laboratories; 5000112). For each sample assessed, 30µg of total protein was loaded onto 10% TGX FastCast Acrylamide gels (Bio-Rad; Cat#: 1610183), ran using Tris/Glycine/SDS buffer (formulated from Bio-Rad Cat#: 161-0732) and imaged using the ChemiDoc XR’s Stain-Free program (Bio-Rad). Resolved proteins were transferred onto PVDF membranes using the Trans-Blot Turbo™ Transfer System at the “Mixed MW” setting (Bio-Rad) and blocked overnight in 5% non-fat powdered milk (w/v) before an overnight incubation with the appropriate primary antibody in TBS buffer with 0.1% Tween-20 (TBST) supplemented with 5% bovine serum albumin (BSA) (w/v). Afterwards, membranes were incubated with species-specific HRP-conjugated secondary antibody (CST; 7074S) for 1 hour prior to using Clarity Western ECL Blotting Substrate (Bio-Rad; Cat#: 170-5060) to visualize protein bands of interest *via* chemiluminescence. For blots that assessed ETC Complex protein expression, images were manually exposed using the Signal Accumulation Mode program to identify the exposure levels of each protein in the antibody cocktail (**Supplementary Figure 4**). Protein band densitometry was analyzed using the methods previously described (31). Briefly, the chemiluminescence intensity of the target bands was normalized to the amount of total protein loaded in its respective lane, as determined by the Stain-Free application on the ChemiDoc XR imager. The target protein expression levels were further normalized relative to their respective control samples and presented as a fold change value. Full scans of each representative blot used in this study can be found in **Supplementary Figures 4**-**7**.

Native protein lysates were collected using M-PER™ Mammalian Protein Extraction Reagent (ThermoFisher; Cat#: 78503) supplemented with Halt™ Protease and Phosphatase Inhibitor Cocktail. The total protein content in each sample was quantified using the DC assay, and based on the data collected, 40µg of total protein was loaded onto 10% TGX FastCast Acrylamide gels and ran using Tris/Glycine buffer (formulated from Bio-Rad Cat#: 161-0734). Resolved protein gels were briefly soaked in Tris-Glycine Transfer Buffer (ThermoFisher; Cat#: LC3675) with 0.1% SDS to improve the transfer process before using Tris-Glycine Transfer Buffer (without SDS) to transfer proteins onto PVDF membranes using the Trans-Blot Turbo™ Transfer System at the “Mixed MW” setting. Afterwards, the gels were incubated in 10% acetic acid (in ddH_2_O) for 15 mins to fix the native proteins to the PVDF membrane. The membrane was then airdried overnight at 4°C before rehydrating the membrane with methanol for 30 seconds. The membrane was rinsed with ddH_2_O prior to blocking overnight with 5% non-fat powdered milk (w/v) in TBST. Subsequent immunodetection was done as described in the previous paragraph. Full scans of each representative blot used in this study can be found in **Supplementary Figures 6**-**10**.

### Extracellular flux analysis via seahorse

RAW264.7 cells were plated onto Seahorse XFp cell culture miniplates (Agilent Technologies) (6x10^3^ cells/well for non-plasmid transfected conditions; 3x10^3^ cells/well for plasmid transfected conditions) and treated as described above (See “siRNA Knockdown of TRNT1” for treatment protocol of non-plasmid transfected conditions using 0.467nM siC and 0.1167nM of each siT per well as well as “Impact of TSPO ligand activation and TSPO overexpression on RAW264.7 function” for treatment protocol of plasmid transfected conditions using 0.06µg pTSPO per well). After LPS or Poly (I:C) treatment, cell culture media was exchanged with Seahorse XF base medium without phenol red (Agilent; Cat#: 103335-100) (pH to 7.4) supplemented with 10mM glucose (ThermoFisher; Cat#: A24940-01), 2mM glutamine (ThermoFisher; 25030-081), and 1mM pyruvate (ThermoFisher; Cat#: 11360-070) and allowed to sit in a non-CO_2_ incubator for 45 mins to 1 hour before the extracellular acidification rate (ECAR) and oxygen consumption rate (OCR) of each condition were measured using a XFp Seahorse Flux Analyzer (Agilent Technologies). Differences in mitochondrial function were evaluated using the Seahorse XFp Cell Mito Stress Test kit protocol. OCR and ECAR measurements were recorded after LPS- and HMW-activated cells were exposed to successive injections of Oligo, FCCP, and ROT/AA. Raw OCR and ECAR values were normalized using total protein levels in each well as determined using the DC assay.

### Statistical analyses

**Figure 1:**
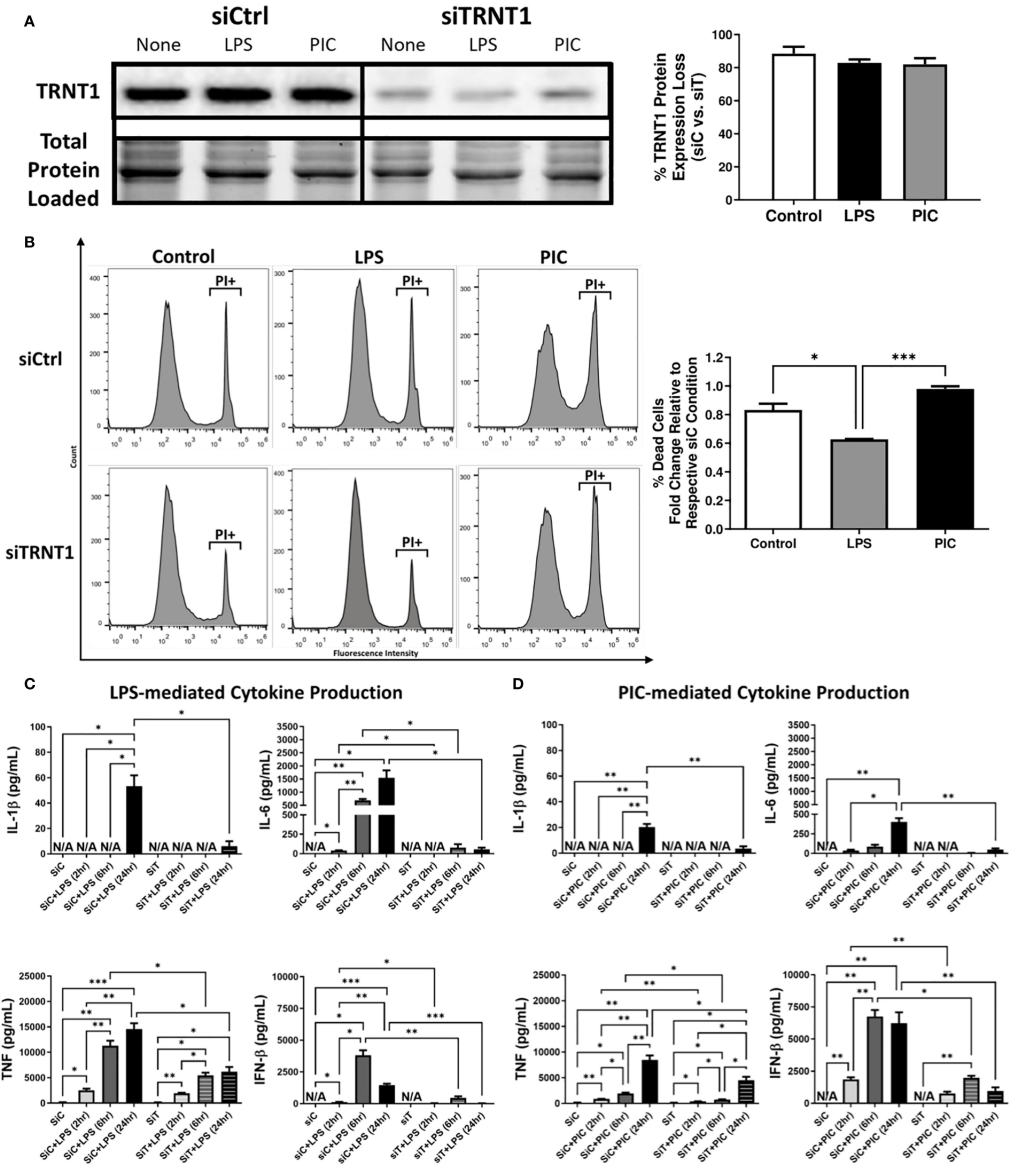
TRNT1 knockdown severely impairs macrophage inflammatory response. RAW264.7 cells were transfected with either a negative control siRNA (siCtrl or siC) or a siRNA cocktail against TRNT1 (siTRNT1 or siT) for 2 days before being treated with 100ng/mL LPS or 10µg/mL Poly (I:C) for 2, 6 and 24 hours. TRNT1 expression was analyzed by immunoblotting after 24 hours. All samples were loaded onto a single gel and cropped to present the observed samples. Protein band intensity was normalized to the total protein levels in their respective lane within their respective gel using the Bio-Rad Stain-Free Application **(A)**. Propidium iodide (PI) staining assessed cell death following 24-hour treatment with either LPS or Poly (I:C) **(B)**. Cytokine secretion profiles of IL-1β, IL-6, TNF, and IFN-β were evaluated in the cell culture supernatant over 24 hours during LPS **(C)** or Poly (I:C) **(D)** activation *via* ELISA. Data represents mean ± SEM of five individual experiments (*p < 0.05, **p < 0.01, and ***p < 0.001).

**Figure 2:**
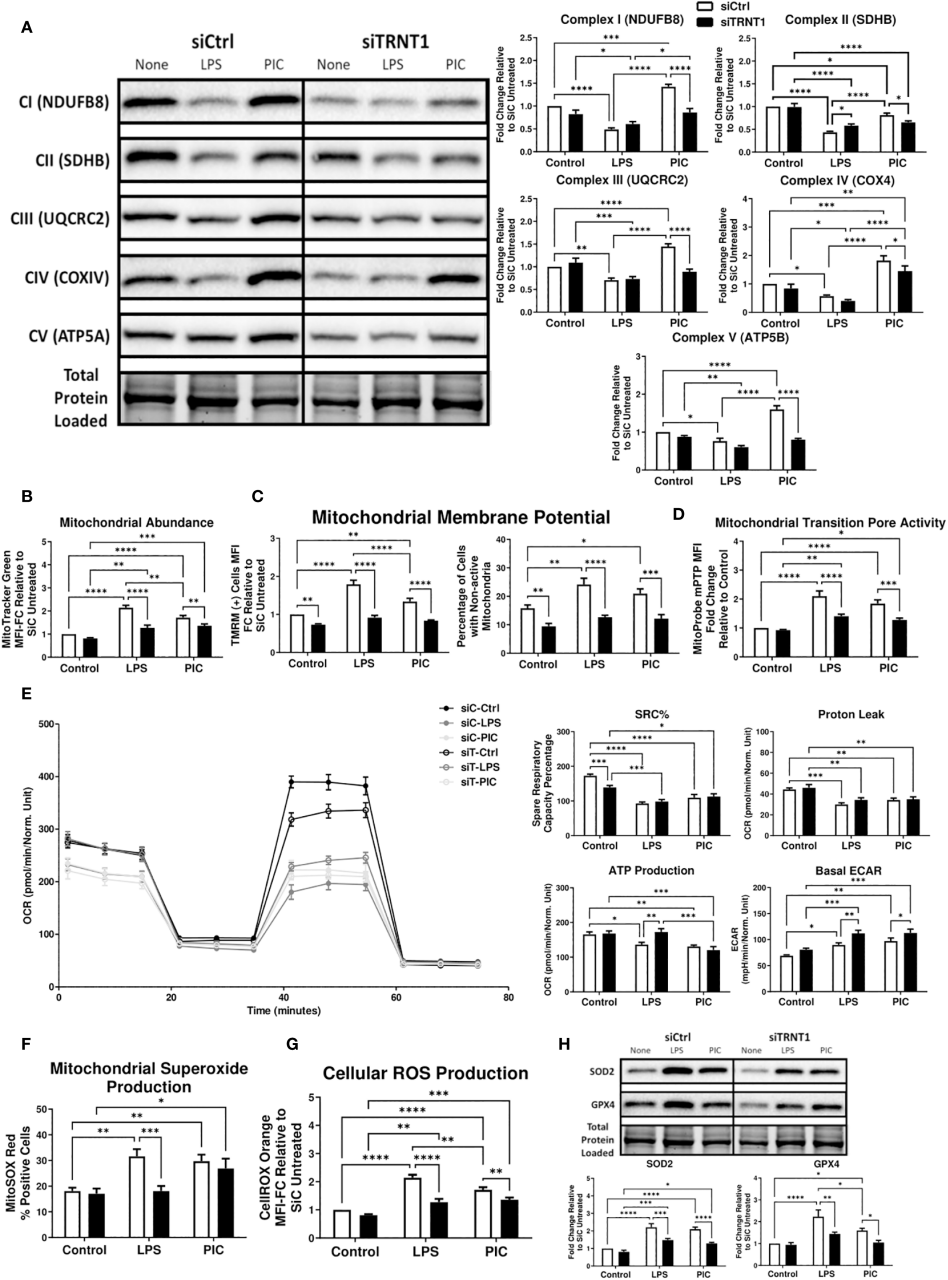
Loss of TRNT1 expression is associated with reduced dynamic mitochondrial reprogramming during TLR engagement. RAW264.7 cells were transfected with siCtrl or siTRNT1 for 2 days prior to TLR engagement with either LPS or Poly (I:C). ETC core protein expression was analyzed by immunoblotting, with representative blot shown on the left and quantified data on the right **(A)**. Changes in mitochondrial membrane potential (MMP) **(B)**, mitochondrial abundance **(C)** and mPTP activation **(D)** were assessed using Tetramethylrhodamine (TMRM), MitoTracker™ Green, and Calcein AM (from Image-IT™ LIVE Mitochondrial Transition Pore Assay Kit) staining, respectively. OXPHOS activity was assessed using the Mito Stress kit, with consecutive Oligomycin (Oligo), Carbonyl cyanide-p-trifluoromethoxyphenylhydrazone (FCCP), and Rot/AA injections. This allowed for the quantification of OXPHOS features such as spare respiratory capacity percentage (SRC%), proton leak, and ATP production as well as basal glycolytic activity by quantifying the extracellular acidification rate (ECAR) **(E)**. Mitochondrial superoxide and Cellular ROS levels were measured using MitoSOX™ Red **(F)** and CellROX™ Orange **(G)** respectively. Antioxidant protein levels (SOD2 & mtGPX4) were quantified using immunoblotting **(H)**. All samples on each blot in this figure were loaded onto a single gel and cropped to present the observed samples. In all blots, band intensity was normalized to the total protein levels in each of their respective lanes within their respective gel using the Bio-Rad Stain-Free Application. Data represents mean ± SEM of five individual experiments (*p < 0.05, **p < 0.01, and ***p < 0.001).

**Figure 3:**
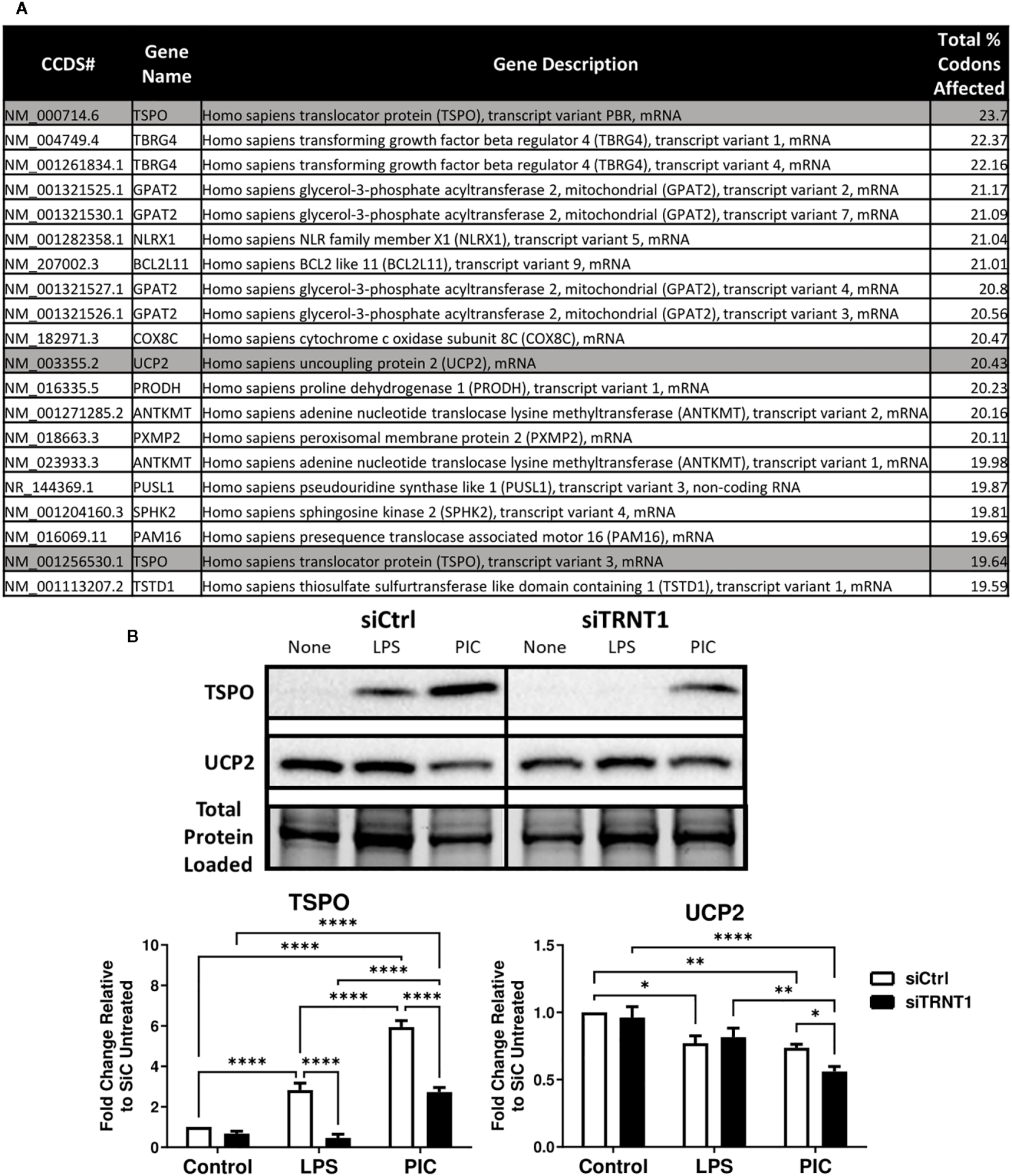
TSPO mRNA transcripts are one of the most affected due to the loss of TRNT1 function. A Perl script using the NCBI human reference genome identified the top 20 mitochondrial-related mRNA transcripts most affected by TRNT1 loss based on key codons of interest, with TSPO and UCP2 highlighted in gray **(A)**. Expression levels of TSPO and UCP2 in RAW264.7 cells transfected with siCtrl or siTRNT1 and subsequently treated with LPS or Poly (I:C) were measured using immunoblotting, with the representative blot shown on the top and quantified data on the bottom **(B)**. All samples on each gel in this figure were loaded onto a single blot and cropped to present the observed samples. In all blots, band intensity was normalized to the total protein levels in each of their respective lanes within their respective gel using the Bio-Rad Stain-Free Application. Data represents mean ± SEM of five individual experiments (*p < 0.05, **p < 0.01, and ***p < 0.001).

**Figure 4:**
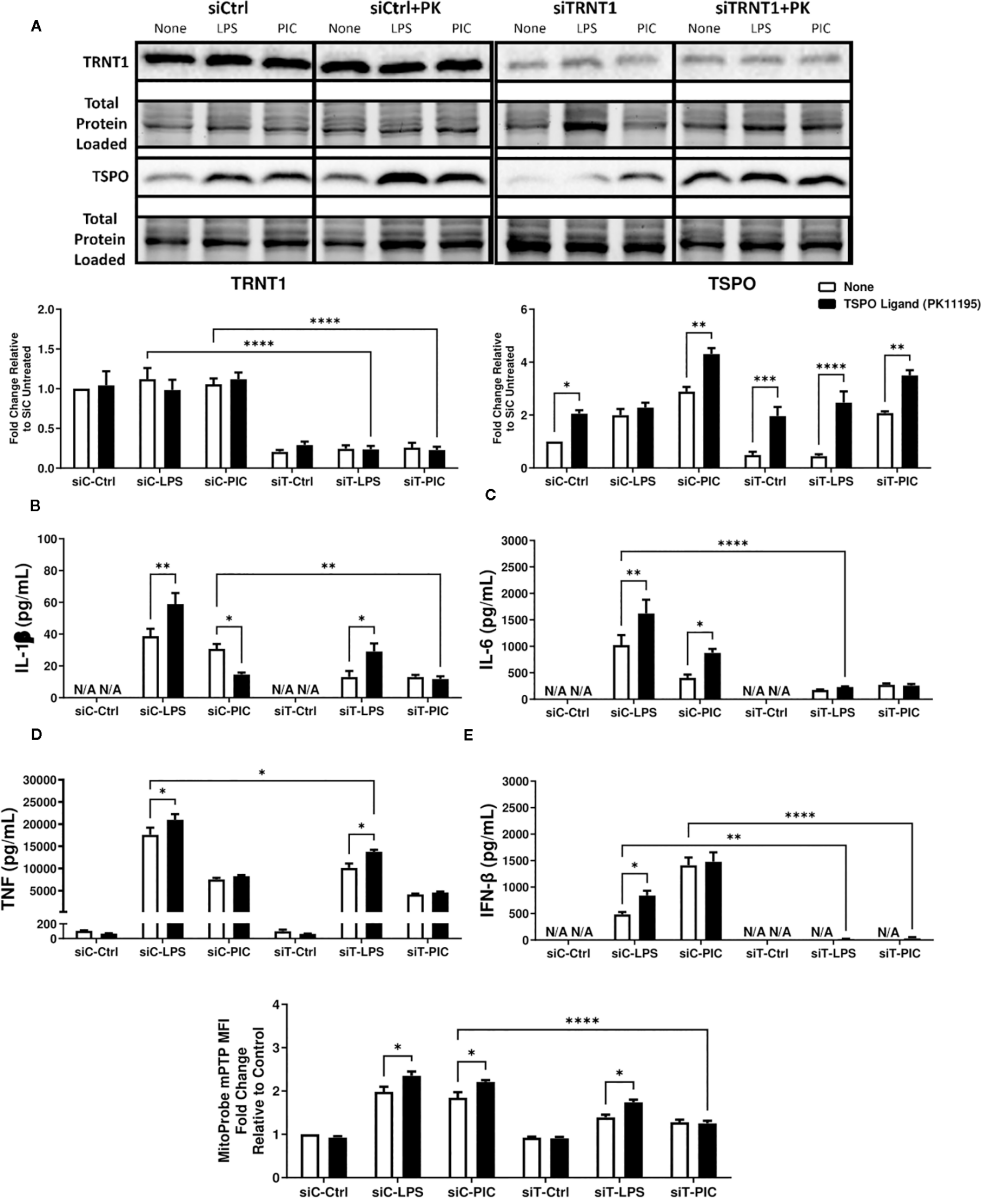
PK11195 may boost TSPO expression but cannot restore siTRNT1-mediated reduction in inflammatory cytokine production. To examine the role of TSPO in macrophage inflammatory responses, RAW264.7 cells were transfected with siCtrl (siC) or siTRNT1 (siT) for 2 days before co-treatment with 10nM PK11195 and either LPS or Poly (I:C). TRNT1 and TSPO protein levels in each condition were assessed via immunoblotting, with the representative blot shown on the top and quantified data on the bottom **(A)**. All siC and siT samples shown were loaded onto separate Stain-Free gels and presented and cropped as one image. The band intensity was normalized to the total protein levels in each of their respective lanes within their respective gel using the Bio-Rad Stain-Free Application. Inflammatory cytokine expression (IL-1β **(B)**, IL-6 **(C)**, TNF **(D)**, IFN-β **(E)**) was evaluated *via* ELISA. mPTP activity was assessed using the Image-IT™ LIVE Mitochondrial Transition Pore Assay Kit **(F)**. Data represents mean ± SEM of five individual experiments (*p < 0.05, **p < 0.01, and ***p < 0.001).

**Figure 5:**
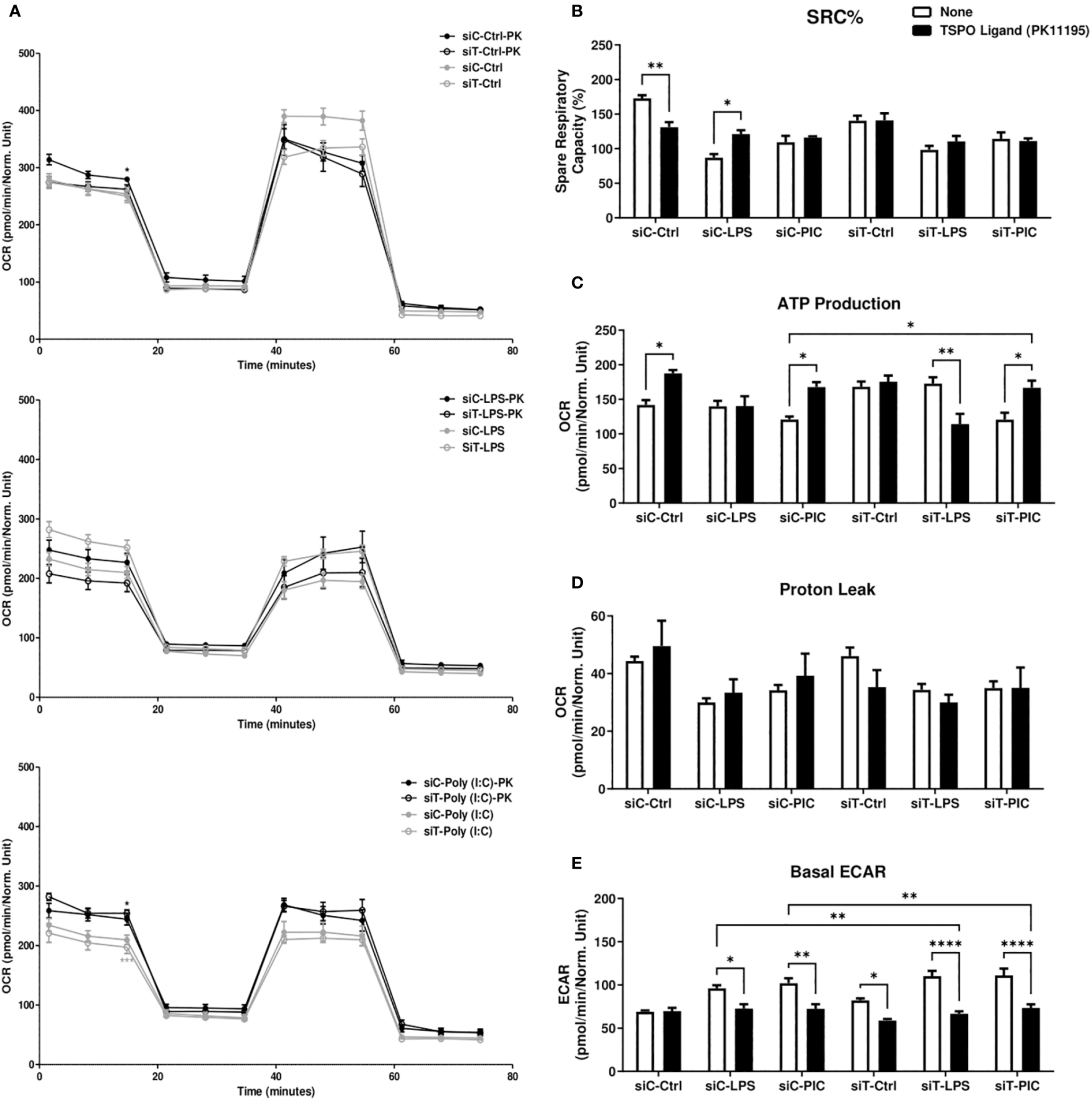
PK11195 increases macrophage mitochondrial dependency during LPS or Poly (I:C) activation. RAW264.7 cells transfected with siC or siT for 2 days were then treated with LPS or Poly (I:C) stimulation and assessed for changes in cellular energetics *via* the Mito Stress test **(A)**. Spare respiratory capacity percentage (SRC%) **(B)**, proton leak **(C)**, and ATP production **(D)** as well as basal glycolytic activity measured *via* ECAR **(E)** were measured *via* Seahorse efflux analysis. Significance in time-course plots **(A)** on the left-side of the figure represents pairwise comparisons between the siC condition and siC+PK condition (Black stars) as well as the siT condition and siT+PK condition (Grey stars). Data represents mean ± SEM of five individual experiments (*p < 0.05, **p < 0.01, and ***p < 0.001).

**Figure 6:**
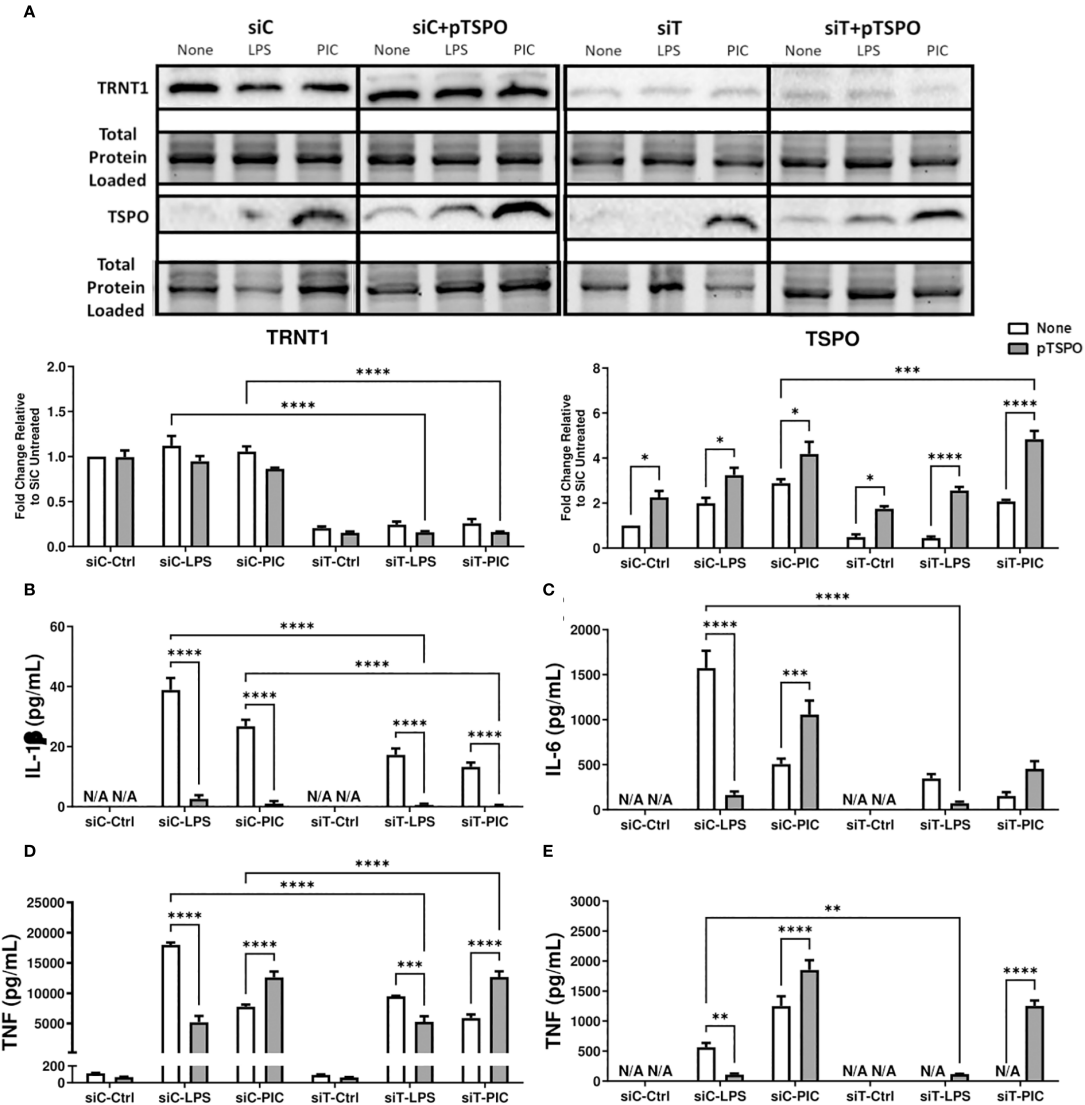
Overexpression of TSPO restores siTRNT1-mediated reduction in inflammatory cytokine production in Poly (I:C)-stimulated macrophages. RAW264.7 cells were either not transfected or transfected with a pCMV6-Entry_TSPO vector (pTSPO) for 24 hours prior to siC or siT transfection for 2 days. Afterwards, the cells were treated with either LPS or Poly (I:C) to examine the role of TSPO in macrophage inflammatory responses. TRNT1 and TSPO protein levels in each condition were assessed *via* immunoblotting, with the representative blot shown on the top and quantified data on the bottom **(A)**. All siC and siT samples shown were loaded onto separate Stain-Free gels and presented and cropped as one image. The band intensity was normalized to the total protein levels in each of their respective lanes within their respective gel using the Bio-Rad Stain-Free Application. Inflammatory cytokine expression (IL-1β **(B)**, IL-6 **(C)**, TNF **(D)**, IFN-β **(E)**) was evaluated *via* ELISA. Data represents mean ± SEM of five individual experiments (*p < 0.05, **p < 0.01, and ***p < 0.001).

**Figure 7:**
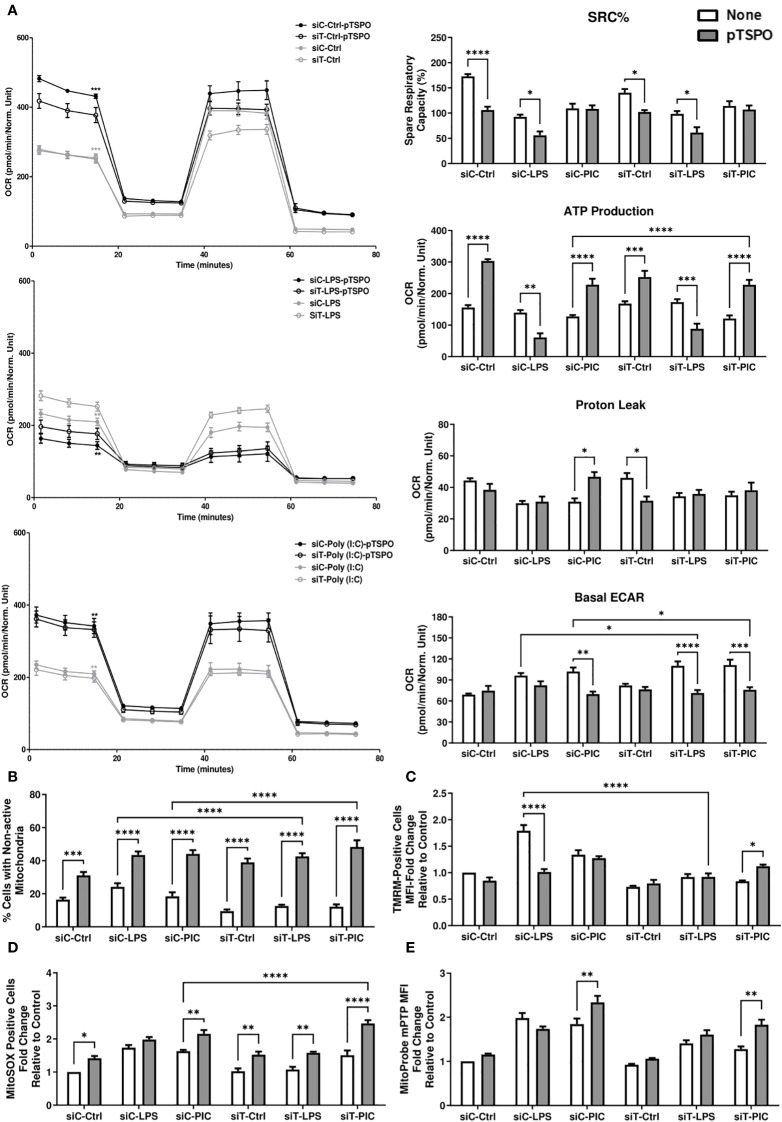
TSPO-mediated restoration of Poly (I:C)-driven inflammation is associated with increased mitochondrial function. RAW264.7 cells with or without pTSPO were transfected with siC or siT for 2 days before siC and siT transfection and subsequent LPS or Poly (I:C) stimulation. Cellular bioenergetics were assessed using the Mito Stress test. Spare respiratory capacity percentage (SRC%), proton leak, and ATP production as well as basal glycolytic activity (measured using ECAR) were quantified *via* Seahorse efflux analysis **(A)**. MMP **(B)**, mitochondrial abundance **(C)**, mitochondrial superoxide **(D)**, and mPTP activity **(E)** were evaluated using TMRM, MitoTracker™ Green, MitoSOX™, and the Image-IT™ LIVE Mitochondrial Transition Pore Assay Kit, respectively. Significance in time-course plots **(A)** represents pairwise comparisons between the siC condition and siC+pTSPO condition (Black stars) as well as the siT condition and siT+pTSPO condition (Grey stars). Data represents mean ± SEM of five individual experiments (*p < 0.05, **p < 0.01, and ***p < 0.001).

**Figure 8:**
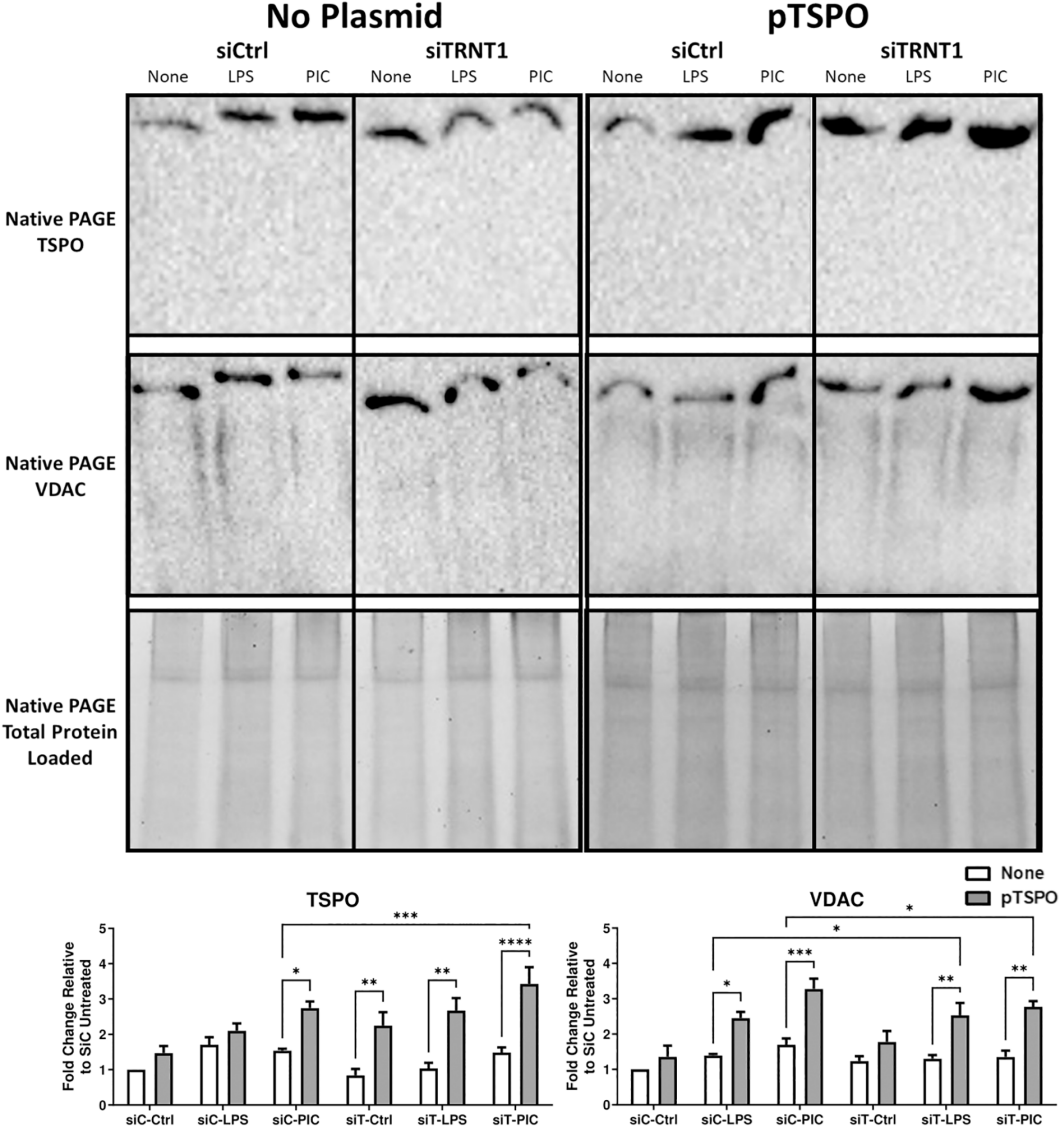
Overexpression of TSPO is associated with increased VDAC recruitment onto the mPTP. RAW264.7 cells that were transfected with or without pTSPO were further transfected with either siCtrl or siTRNT1 before treatment with LPS or Poly(I:C). Afterwards, total cell lysates were harvested under a Native PAGE protocol. Native TSPO and VDAC protein expression were examined using immunoblotting, with the representative blot shown on the top and quantified data on the bottom. All non-plasmid and plasmid-containing samples were loaded onto separate Stain-Free gels and presented and cropped as one image. The band intensity was normalized to the total protein levels in each of their respective lanes within their respective gel using the Bio-Rad Stain-Free Application. Data represents mean ± SEM of four individual experiments (*p < 0.05, **p < 0.01, and ***p < 0.001).

Data used in this study was analyzed using GraphPad Prism software (version 10.4.1). The values shown represent the mean ± SEM of biological replicates, where the number of replicates is reported in the figure legends. Statistical significance was calculated using either a one-way ANOVA with post tests (**Figure 1** and **Supplementary Figures 1**, **3**) or a two-way ANOVA with post tests (**Figures 2**–**8** and **Supplementary Figure 2**) (*p < 0.05, **p < 0.01, ***p < 0.001, and ****p < 0.0001).

## Results

### Loss of TRNT1 results in a severe reduction in inflammatory cytokine production

Given the link between SIFD and inflammatory episodes (5, 13–15), we first characterized the changes in inflammatory cytokine expression associated with TRNT1 deficiency in macrophages. First, we assessed multiple macrophage models systems (mouse cell line RAW264.7, mouse primary bone marrow derived macrophages (BMDM), and human cell line THP-1 [differentiated with 20ng/mL Phorbol 12-myristate 13-acetate (PMA) or left undifferentiated]) to identify the best model system to conduct transfection experiments. Under similar conditions, RAW264.7 and undifferentiated THP-1 cells achieve the highest transfection efficiency (87.7% and 77.6% respectively; **Supplementary Figure 1A**). But, allowing undifferentiated THP-1 cells to sit 2- or 3-days post transfection (the duration of time for downstream treatments done in this study) significantly reduced transfection efficiency (**Supplementary Figure 1B**). As a result, for the remainder of this study, we used RAW264.7 cells to conduct experiments. Following transfection with RAW264.7 cells, they were treated with either 100 ng/mL lipopolysaccharide (LPS; to mimic antibacterial responses) or 10 µg/mL high molecular weight polyinosinic-polycytidylic acid (Poly (I:C); to mimic antiviral responses) for 24 hrs. These two agonists were chosen due to their frequent use to investigate differential innate immune activation states (32). TRNT1 expression was significantly reduced by 80-90%, similar to levels seen in SIFD patients (6), with no significant differences between cells treated with LPS or Poly (I:C) (**Figure 1A**). To ensure that this degree of knockdown did not equate to any loss in cell viability, we stained the cells with the cell death marker Propidium iodide (PI). We found that siTRNT1 transfection did not increased cell death in any treatment condition (**Figure 1B**). In fact, siTRNT1 cells stimulated with LPS were found to have decreased cell death compared to their siCtrl counterparts (**Figure 1B**). We then examined the inflammatory output of these macrophages by measuring the secreted levels of IL-1 β, IL-6, IFN- β and TNF, key cytokines produced during bacterial and viral challenges (21, 33). As expected (28), LPS stimulation in siCtrl-treated cells produced higher levels of inflammatory cytokines compared to Poly (I:C) stimulation except for IFN-β secretion (**Figures 1C, D**). In contrast, TRNT1 knockdown resulted in a near complete loss in IL-1β, IL-6, and IFN-β secretion as well as a ~50% reduction in TNF expression in both LPS and Poly (I:C) treatments (**Figures 1B–E**) (LPS: IL-1β - ↓88.76%, IL-6 - ↓96.49%, IFN-β - ↓98.40% and TNF - ↓58.58%; Poly (I:C): IL-1β - ↓83.10%, IL-6 - ↓88.08%, IFN-β - ↓84.66% and TNF - ↓48.41%). Interestingly, the trends seen in the cytokine profiles of LPS and Poly (I:C) over 24 hours are maintained in siTRNT1 cells, but at a much lower magnitude compared to the siCtrl cells. Furthermore, we examine if this effect was seen across different TLR agonists by testing a panel of TLR agonists known to interact with different TLRs and/or induce differential responses at the same receptor (34, 35): LPS (TLR4), High molecular weight Poly(I:C) (HMW PIC; TLR3), Low molecular weight Poly(I:C) (LMW PIC; TLR3), Imiquimod (R837; TLR7), Pam2CGDPKHPKSF (FSL-1; TLR2/6), E.coli ssDNA (EC ssDNA; TLR9), and ssRNA40 (TLR9) (**Supplementary Figure 2A**) We were able to confirm that TRNT1 KD resulted in a similar loss in inflammatory capacity (**Supplementary Figures 2B, C**). To verify that these effects were due to the loss of TRNT1 protein and not any off-target effects of siRNA, we conducted TRNT1 rescue experiments by transfecting RAW264.7 cells with pTRNT1 following siTRNT1 knockdown but prior to LPS or Poly (I:C) stimulation. Not only did the use of pTRNT1 recover TRNT1 protein levels to that seen in the siCtrl cells (**Supplementary Figure 3A**), but TRNT1 rescue recovered most inflammatory cytokine production (**Supplementary Figures 3B, C**). This indicates that TRNT1 deficiency was associated with a deficit in the inflammatory capacity of RAW264.7 cells.

### TRNT1 knockdown is linked to a reduction in dynamic mitochondrial function

Due to TRNT1’s localization to the mitochondria (36), previous research linking its expression to alterations in mitochondrial function (13, 15), and the importance of mitochondria in driving an immune cell’s inflammatory response (22–27), we next investigated whether the loss of TRNT1 affected the mitochondrial reprogramming required for facilitating an immune response. First, we assessed changes in the expression of ETC complexes by comparing the levels of key core proteins after TRNT1 knockdown. As previously reported by our group (28), siCtrl cells stimulated with LPS and Poly (I:C) drove differential reassembly of their ETC complexes. LPS-stimulated cells have reduced expression of all five ETC complexes, whereas Poly (I:C) stimulation significantly increased the expression of all complexes except for Complex II which was significantly reduced (**Figure 2A**), suggesting that both LPS and Poly (I:C) stimulation resulted in differential reprogramming of their respective ETC architecture. To link these changes to mitochondrial function, we next assessed differences in mitochondrial abundance *via* MitoTracker™ Green, alterations in mitochondrial membrane potential (MMP) using TMRM, and changes in mPTP activity using the Image-IT™ LIVE Mitochondrial Transition Pore Assay kit. While treatment with both TLR ligands resulted in differential changes in the ETC architecture, there was an upregulation in mitochondrial abundance for siCtrl cells after treatment with both ligands, with a more pronounced upregulation in LPS-stimulated cells compared to Poly (I:C)-stimulated cells (**Figure 2B**). These changes in mitochondrial abundance were associated with a comparable fold increase in the magnitude of MMP in active mitochondria as well as a significant increase in the percentage of cells with low TMRM sequestration (**Figure 2C**). In addition, mPTP activity in both treatments was also elevated similarly to that seen with mitochondrial abundance (**Figure 2D**). After TRNT1 knockdown, the ETC complex expression during LPS stimulation remained unchanged except for a minor increase in Complex II expression (**Figure 2A**). In contrast, the ETC complexes that were upregulated in siCtrl cells stimulated with Poly (I:C) were significantly diminished after TRNT1 knockdown (**Figure 2A**). Further, TRNT1 knockdown was associated with a reduction in mitochondrial abundance, MMP levels in active mitochondria, cells possessing non-active mitochondria and mPTP activity to that seen in siCtrl cells treated with LPS and Poly (I:C) (**Figures 1B–D**), suggesting a decrease in mitochondrial abundance and function in the absence of TRNT1.

To link these changes to the bioenergetic capabilities of mitochondria, we next used the Cell Mito Stress Test kit (Agilent) to assess specific differences in oxidative phosphorylation (OXPHOS). Features of OXPHOS activity were calculated based on changes in OCR in response to successive injections of Oligo, FCCP and ROT/AA. Consistent with the literature (28, 37–39), LPS and Poly (I:C) stimulations in siCtrl cells resulted in reduced spare respiratory capacity percentage (SRC%), ATP production, proton leak and increased basal ECAR, which allowed macrophages to compensate for reduced mitochondrial energy production (22, 40, 41) (**Figure 2E**). In the absence of TRNT1, there were no significant changes in SRC% and proton leak in LPS and Poly (I:C)-stimulated cells, but there was a further amplification of the level of basal ECAR compared to siCtrl cells (**Figure 2E**). Interestingly, only LPS-stimulated RAW264.7 cells exhibited a restoration of ATP production over their respective siCtrl counterpart (**Figure 2E**). Overall, this indicated that the loss of TRNT1 alters the ETC architecture and mitochondrial structural dynamics in such a manner that it affects key functions such as MMP, and mPTP activity during a macrophage effector response.

### Loss of TRNT1 differentially affects macrophage ROS levels

A byproduct of OXPHOS activity is the release of mtROS due to electron leakage from Complexes I and III (42, 43). While the high-level generation of mtROS may be damaging to the cell if redox balance is not maintained, low levels of ROS can regulate several cellular processes, including cell proliferation, differentiation and migration (44, 45). Given the intrinsic link between TRNT1 and alterations in mitochondrial function (13, 15), we investigated whether ROS production is altered in the absence of TRNT1. First, we quantified mitochondrial superoxide and cellular ROS production using the fluorescent probes MitoSOX™ Red and CellROX™ Orange, respectively. In siCtrl cells, both LPS and Poly (I:C) stimulation significantly increase mitochondrial superoxide and cellular ROS production compared to the untreated condition (**Figures 2F, G**). Reducing TRNT1 expression results in a reduction of both mitochondrial and cellular ROS in LPS-stimulated cells, but while cellular ROS was also diminished in Poly (I:C)-stimulated cells, mitochondrial superoxide production was sustained (**Figures 2F, G**). When examining the antioxidant response of siCtrl cells, both TLR ligands significantly induced the expression of superoxide dismutase 2 (SOD2) and glutathione peroxidase 4 (GPX4) as a protective mechanism to limit ROS-associated damage (**Figure 2H**). In contrast, TRNT1 knockdown abrogated the induction of both antioxidant proteins (**Figure 2H**). This highlights that while the antioxidant response may be diminished in the absence of TRNT1, only Poly (I:C) engagement can induce comparable levels of mtROS, which did not equate to any purposeful immune response observed given the importance of mtROS to Poly (I:C)-mediated macrophage effector function (28).

### TSPO and UCP2 expression is affected and altered due to the loss of TRNT1

We previously shown that TRNT1 deficiency is associated with a decreased abundance of tRNA pools and altered expression of distinct proteins (16). We were therefore curious to identify additional mitochondrial genes disproportionately affected by the loss of TRNT1. We developed a Perl script that was incorporated into BioPerl to calculate the percentage of key codons found in all human mRNA transcripts that were most affected by TRNT1-causing mutations in SIFD patient fibroblasts (16). This list was further filtered down to identify all transcripts from mitochondrial-related genes based on the updated MitoCarta 3.0 database (30) and sorted in descending order of their codon abundance (**Supplementary Table 1**). To identify putative transcripts that were differentially affected by TRNT1 deficiency, we set a threshold at the percentage point 2 standard deviations higher than the mean percentage of all transcripts (Mean = 12.603 ± 2.282; p<0.05). Based on that criterion, ninety-nine distinct mitochondrial-related mRNA transcripts from sixty-four different genes were identified (**Supplementary Table 1**). Among the top 20 differentially affected mRNA transcripts, two genes with clear links to inflammatory processes stood out: the Translocator protein (TSPO) (46, 47) and Uncoupling protein 2 (UCP2) (48–50) (**Figure 3A**). These proteins were selected for further analysis in our system.

Interestingly, the expression of both proteins was differentially affected by the loss of TRNT1. In siCtrl-transfected cells, both TLR ligands significantly induced TSPO expression, whereas UCP2 expression was significantly diminished (**Figure 3B**). In siTRNT1-transfected cells, TSPO expression was only induced during Poly (I:C) stimulation but to a lesser degree, whereas untreated and LPS-treated cells displayed minimally detectable levels of TSPO (**Figure 3B**). TSPO expression was also lost in other TLR agonists known to induce a robust inflammatory response (FSL-1, R837) (51, 52) (**Supplementary Figures 2B, C**). Regarding UCP2 expression, it was further reduced in Poly (I:C)-stimulated cells, whereas both control and LPS-stimulated cells maintained similar levels of expression to their respective siCtrl counterparts (**Figure 3B**). However, the observed UCP2 expression patterns did not match the changes seen in the proton leak data given its role as a dicarboxylate/proton transporter on the mitochondrial inner membrane (45, 53) (**Figure 2E**). As such, TSPO represented an attractive target for further analysis.

### TSPO ligand may boost respiration but does not recover macrophage effector function

The outer mitochondrial membrane protein TSPO is closely associated with other mitochondrial channel proteins/complexes such as VDAC and Adenine nucleotide translocase (ANT) of the mPTP (54–56) and is linked to various mitochondrial functions ranging from cholesterol import, regulation of MMP, oxidative stress, mitochondrial metabolism, inflammation and apoptosis (46, 47, 54, 57–64). Due to its links to mitochondrial function, inflammation, and its ability to be modulated by TRNT1, we explored whether restoring TSPO function would help recover the diminished macrophage function in siTRNT1 cells.

First, we characterized the expression levels of TRNT1 and TSPO in the presence or absence of TSPO ligand PK11195, which had been shown to stabilize TSPO structure, increase TSPO levels/activity and to promote mPTP opening (48, 51, 56). To do so, we first transfected RAW264.7 cells with either siCtrl or siTRNT1 before co-treating them with PK11195 and either LPS or Poly (I:C). We found that in siCtrl-transfected cells, PK11195 did not significantly affect TRNT1 expression in all conditions but caused increased TSPO expression in untreated and Poly (I:C)-treated cells but not LPS-treated cells (**Figure 4A**). In siTRNT1-transfected cells, while TRNT1 expression remained suppressed, TSPO protein levels stabilized in the presence of PK11195 (**Figure 4A**). In the siCtrl-transfected cells, the presence of PK11195 caused increased inflammatory cytokine expression during LPS stimulation, while Poly (I:C) stimulation led to decreased IL-1β, increased IL-6 production, and no change in TNF or IFN-β secretion (**Figures 4B–E**). Interestingly, this increase in TSPO levels did not result in any significant recovery of inflammatory cytokine production in either LPS or Poly (I:C) treatment in siTRNT1-transfected cells, though LPS-stimulated cells did produce slightly higher levels of IL-1β and TNF compared to cells not exposed to PK11195 (**Figures 4B–E**). Conditions that saw significant changes in inflammatory cytokine expression were associated with increased mPTP activity (**Figure 4F**). When we examined cellular bioenergetics, we saw that PK11195 reduced basal ECAR levels to that of the untreated siCtrl cells (**Figure 5E**) while both untreated and Poly (I:C)-treated cells did see small increases in basal respiration and ATP production after PK11195 co-treatment but not proton leak (**Figures 5A–D**). This suggested that TSPO ligand stabilization/activation reduces the glycolytic demand on RAW264.7 cells and increases mitochondrial energy reliance. But when TRNT1 levels were reduced, only LPS-stimulated cells saw a modest increase in inflammatory cytokine production while PK11195 was not sufficient to recover the inflammatory cytokine expression in Poly (I:C)-stimulated cells.

### TSPO overexpression restores siTRNT1-mediated reduction of inflammatory cytokine production

We inquired whether increasing the expression of functional TSPO before siRNA transfection was protective against the reduction in inflammatory cytokine production. We transfected RAW264.7 cells with pTSPO before siTRNT1 knockdown and subsequent LPS or Poly (I:C) stimulation. Similar to PK11195 treatment, pTSPO transfection resulted in increased TSPO expression in all conditions, even in siTRNT1 transfected cells (**Figure 6A**). Intriguingly, LPS and Poly (I:C) stimulation in the presence of pTSPO resulted in different cytokine expression profiles. While the expression of all inflammatory cytokines was significantly diminished in LPS-stimulated cells irrespective of TRNT1 gene silencing, Poly (I:C) stimulation boosted inflammatory cytokine production across all conditions except for IL-1β expression (**Figures 6B–E**). In fact, siTRNT1 cells transfected with pTSPO showed similar inflammatory output to that observed in non-pTSPO transfected siCtrl cells, including increased TNF secretion (**Figures 6B–E**). This suggests that the insertion of functional TSPO into the mitochondria before the loss of TRNT1 function may be sufficient to prevent any potential reduction of inflammatory capacity.

Next, we attempted to link this recovery of inflammatory function to any alterations in mitochondrial function. We observed that pTSPO-transfected LPS-stimulated cells had further reduced respiration, SRC%, and ATP production regardless of their TRNT1 status (**Figure 7A**). In contrast, Poly (I:C)-stimulated cells had increased respiration, ATP production, and reduced basal ECAR when TSPO was overexpressed (**Figure 7A**). While all conditions were characterized by more cells with nondetectable TMRM signal due to the overexpression of TSPO (**Figure 7B**), the decrease in respiration seen in LPS-stimulated cells was linked with low MMP relative to non-transfected siCtrl-LPS-treated cells (**Figure 7C**). Conversely, Poly (I:C)-stimulated cells transfected with pTSPO maintained comparable TMRM signal, including when TRNT1 levels are reduced (**Figures 7B, C**). This was also associated with a further amplification of mtROS production in both siCtrl and siTRNT1 cells (**Figure 7D**). In addition, Poly (I:C)-stimulated cells exhibited increased mPTP activity after TSPO overexpression irrespective of TRNT1 status such that the pTSPO-SiT-PIC condition had mPTP activity comparable to that seen in non-pTSPO transfected siCtrl Poly (I:C) stimulated cells (**Figure 7E**). This shows that the overexpression of TSPO drove increased mitochondrial function and dependency in siCtrl cells, which is detrimental to the glycolytically-supported LPS response but augments the mitochondrial-dependent Poly (I:C) response. Further, this shift in pTSPO-associated bioenergetics may be sufficient in restoring the effector response of Poly (I:C)-treated siTRNT1 cells.

Finally, given TSPO’s relationship to the mPTP complex, we examined the differences in the interactions between TSPO and VDAC, a core mPTP complex protein, using Native-PAGE Western blots. We found that during Poly (I:C) stimulation, the TSPO overexpression was associated with increased recruitment of VDAC onto the mPTP in both siCtrl and siTRNT1 cells (**Figure 8**). The same was also evident in LPS-stimulated cells that overexpressed TSPO (**Figure 8**). This not only identified TSPO as a central player in maintaining proper mitochondrial function but maintaining functional TSPO protein may be critical to a proper macrophage effector response against viral ligands such as Poly (I:C) or viral pathogens.

## Discussion

SIFD is an autosomal recessive syndrome caused by mutations in TRNT1, a key enzyme of tRNA production and maturation (4–8). The hallmark SIFD pathologies include sideroblastic anemia, recurring fevers and autoinflammatory episodes, developmental delay, deficiencies in mature B cells and serum Ig levels (5, 13–15). As more atypical or mild SIFD cases are identified worldwide, it is increasingly recognized that alterations in TRNT1 expression/function may be associated with more heterogenous pathologies and increased susceptibilities to bacterial and viral infections such as *Staphylococcus*, cytomegalovirus, & COVID-19 (5, 65–70). Some SIFD patients present with ataxia, severe hearing loss, retinitis pigmentosa, seizures, and cardiomyopathy to name a few (4). Given this, more research into the wide-ranging effects of TRNT1-related immunodeficiencies is required. We report that the siRNA silencing of TRNT1 impairs acute macrophage inflammatory response treated with LPS or Poly (I:C), due to a lack of mitochondrial reprogramming and limited mitochondrial function. Further, we identified TSPO as a key protein that connects TRNT1 expression to mitochondrial activity and inflammation, which was differentially regulated during LPS and Poly (I:C) stimulation. While using a TSPO ligand was not sufficient to recover the cellular inflammatory responses with reduced TRNT1, TSPO overexpression recovered the inflammatory response of only TRNT1-deficient Poly (I:C)-stimulated cells. This recovery was associated with increased recruitment of VDAC onto the mPTP, leading to increased OXPHOS activity, mtROS production, and mPTP activity. This suggests that TSPO may potentially restore macrophage antiviral immune responses for patients with SIFD.

Over the last decade, researchers have worked to better understand SIFD pathology to improve current therapeutic approaches. As of May 2025, 48 unique mutations have been identified in 65 patients (68–72). The most frequent mutations reported are missense mutations, and while frameshift and non-sense mutations have been reported (60, 62), no patients have been identified with biallelic non-sense or frameshift mutations (73). This suggests that residual TRNT1 activity is required for fetal development. In accordance with this, the complete loss of TRNT1 is lethal in yeast (6). TRNT1 modify the separate pools of cytosolic and mitochondrial tRNAs by adding the CCA trinucleotide to their 3’ end, vital for proper amino acid binding, tRNA positioning within the ribosome complex, and translation termination (4, 8, 74, 75). Other TRNT1 roles include maintaining and repairing tRNA-CCA sequences which are sensitive to cleavage and degradation (16, 75) as well as preventing damaged tRNAs from ribosome incorporation (10). Given this, it is plausible that TRNT1 deficiency can have adverse effects on cell function. Studies have shown that the impaired maturation of nuclear and mitochondrial tRNAs result in a global protein synthesis defect in TRNT1-deficient cells (15, 76). But this is not always the case. Our group has shown that disease-causing mutations in patient-derived fibroblasts do not affect the subcellular localization of TRNT1 nor possess any significant mitochondrial morphological differences compared to healthy controls (13). But two other groups studying patients with separate SIFD-causing mutations found that distinct mitochondrial tRNAs were differentially affected (7, 8), which further complicates the understanding of disorders like SIFD.

Studies have linked SIFD and TRNT1-causing mutations to changes in mitochondrial structure and function, affecting processes such as energy production, redox balance and apoptosis (77–79). We have previously shown that SIFD patient-derived fibroblasts exhibit reduced basal and maximal respirations rates due to decreased expression of key core ETC proteins (NDUFB8, SDHB, COXII) (13), likely resulting from defective TRNT1-mediated mitochondrial translation (7). Other identified metabolic abnormalities include increased levels of alanine, threonine, glycine and branched-chain amino acids in patient plasma (8) as well as increased oxidative stress sensitivity leading to angiogenin-dependent tRNA cleavage (16). These observations suggest that metabolic dysregulation is a central pathological feature of SIFD pathology.

Macrophages undergo metabolic reprogramming to meet the specific bioenergetic and biosynthetic demands to mount an effective and specific effector response as well as recruit other immune cells such as T and NK cells (37, 71–74). This process involves ligand- and pathogen-specific mitochondrial adaptations. For example, the hyperinflammatory LPS-mediated response is associated with impaired OXPHOS function while elevating aerobic glycolysis (Warburg effect) to support rapid energy requirements and cytokine production (22, 37, 80–83). This shift is driven, in part, by blockages along the TCA cycle, and the repurposing of mitochondrial function away from ATP production towards mtROS production to power antibacterial responses *via* reverse electron transport (RET), causing electrons to travel from Complex II back to complex I, which also increases MMP (22, 28, 40, 84). Conversely, Poly (I:C)-driven inflammation requires some level of OXPHOS activity for both energy production and mounting a proper effector response, but still producing high levels of mtROS, *via* increased Complex III expression, to facilitate inflammatory and antiviral signaling (28, 34). Thus, the dynamic remodeling of ETC complexes allow macrophages to modulate cytokine production following TLR engagement. We found that the differential dynamic ETC remodeling during both LPS and Poly (I:C) activations result in increased MMP, mtROS and pore activity, a feature seen in most inflammatory macrophage states because of fission events and cristae expansion, which dampens ETC efficiency and supercomplex formation (85, 86). Further, these crucial mitochondrial adaptations in both activation states were affected by TRNT1 knockdown such that the degree of ETC expression change in both LPS- and Poly (I:C)-stimulated macrophages were attenuated. This led to a severe reduction in the acute inflammatory response as well as diminished MMP, mitochondrial abundance, mPTP activity and mitochondrial antioxidant response. Yet interestingly, siT-transfected Poly (I:C) cells maintain elevated mtROS likely due to electron leakage from a less efficient ETC (no change in ETC flux as indicated by no difference in ATP production and decreased ETC expression). These findings link TRNT1 to macrophage mitochondrial reprogramming and their immune function.

Given Sasarman and colleagues’ study (7) showing that TRNT1 mutations led to defective mitochondrial translation and the clear effects TRNT1 silencing had on altering mitochondrial functions in our study, we investigated whether other mitochondrial proteins were affected by the absence of TRNT1 and identified the outer mitochondrial membrane protein TSPO as a key protein. TSPO, a biomarker of neuroinflammation and microglial activation (46, 47, 62), was initially discovered as a peripheral binding site for benzodiazepines (87) with later work showing that TSPO can bind and transport cholesterol into mitochondria to initiate steroidogenesis (58, 88). TSPO has also been implicated with cellular bioenergetics and other mitochondrial processes (46) due to its association with the mPTP, an apoptosis initiating complex *via* the stimulated opening of non-specific pores across the mitochondrial membranes and the release of apoptosis-inducing molecules including cytochrome c (Cytc), second mitochondria-derived activator of caspases (Smac)/Diablo, apoptosis-inducing factor (AIF), and endonuclease G (58, 61, 89, 90). The mPTP complex comprises VDAC, Bcl-2, Bax, TSPO and hexokinase (HK-I & II) on the outer mitochondrial membrane; creatine kinases (CK) in the intermembrane space; and adenine nucleotide translocator (ANT), Cyclophilin D (CypD) and Complex V in the inner mitochondrial membrane (90). Metabolic pathways can regulate mPTP activity, with glycolysis capable of modulating pore activation *via* HK (91) and the ETC influencing mPTP, either through manipulating Complex V activity, inhibition of Complex I, or modulation of MMP (92–94). Further, CypD overexpression increases MMP, OCR activity, ATP production, and resistance to oxidative stress *via* increased Complex III expression and supercomplex formation due to direct Complex III/CypD interactions (95, 96), further linking the ETC to the mPTP complex.

Coinciding with the literature (97, 98), we found that TSPO expression is induced during LPS and Poly (I:C) stimulation but is differentially regulated following TRNT1 silencing, resulting in differential ETC modulation and reduced mitochondrial function. While the TSPO ligand PK11195 boosted respiration and did not reduce TSPO expression, this was not sufficient to recover the siTRNT1-mediated decline in inflammatory function. PK11195 binds 1:1 to TSPO, stabilizing its structure by bringing its five transmembrane helices closer together (56, 64). However, evidence indicates that PK11195 can be incorporated into lipid bilayers even in the presence of TSPO, altering the rigidity of the membrane (99, 100), potentially causing some of the TSPO-independent effects reported by some groups (59, 101, 102). It is possible that the PK11195-mediated boost in respiration seen in siTRNT1 cells may be due to interactions between PK11195 and the mitochondrial membrane(s) and not due to its binding with TSPO. Given TRNT1’s role in tRNA maturation and protein synthesis, it is possible that PK11195 cannot properly stabilize TSPO due to its misfolding caused by reduced TRNT1 expression and hampering a macrophage’s inflammatory response. This idea aligns with a study from Milenkovic and colleagues (103), where they examined the two most frequently occurring missense mutations of TSPO (A147T, R162H), which were linked to reduced function and possible neurological disorders, and found that they decreased the half-life of the protein by 25%. In addition, the increased flexibility of the mutant structures was associated with decreased protein stability (103). Given that unbound TSPO exists in a dynamic, fluid, partially folded state (104), it is possible that the changes in protein flexibility, whether due to mutations or misfolding events, may affect its ability to interact with its ligands or proteins of the mPTP. Owen and colleagues found that a second generation TSPO ligand (PBR28) is reported to have reduced affinity for A147T-TSPO (105) and thus a better understanding of the interactions between TSPO and its ligands may lead to the development of next generation ligands capable of maintain structure-function relationship of TSPO under disease conditions (106).

Of note, TPSO overexpression before TRNT1 knockdown restored the inflammatory capacity of Poly (I:C)-stimulated cells. This suggests that functional TSPO is not only required for mounting an inflammatory response, but one that is linked to mitochondrial function. This is further evident because LPS-stimulated macrophages, a state that generates its inflammatory cytokines *via* the Warburg effect (107), saw an acute reduction in inflammatory cytokine expression when TSPO was overexpressed. In addition, TSPO overexpression in LPS-stimulated cells further reduced their mitochondrial output without any appreciable gain in compensatory glycolytic function. Given that increased TSPO was associated with decreased MMP in these cells, the presence of more pores may have dissipated the MMP and thus disrupted the RET process, which normally enhances MMP during LPS activation (37, 75).

Conversely, Poly (I:C) stimulation, which relies on OXPHOS and mtROS production for its effector response (28), resulted in amplified inflammatory capacity linked to boosted respiration and mitochondrial function with both PK11195 and TSPO overexpression in siCtrl cells. This is supported by a Djafarzadeh et al. study (108) that showed that Poly (I:C) activation induces mPTP activation, and that its functionality is positively correlated with PIC-induced NF-κB signaling. Further, TSPO overexpression prior to TRNT1 knockdown recovered nearly all inflammatory capacity seen in macrophages with normal TRNT1 levels except for IL-1β. This may be linked to the presence of HK in the mPTP complex, as glycolytic ATP can be a secondary trigger to activate the inflammasome and cleave IL-1β (37, 109). In addition, dissociation of HK from VDAC and the mPTP results in inflammasome assembly and subsequent IL-1β and IL-18 maturation, thus tying HK-derived ATP to IL-1β cleavage. Given this, the decrease in ECAR seen due to TSPO overexpression would suggest less glycolytic ATP is present to trigger IL-1β cleavage. Further, this recovery in pTSPO-linked inflammatory cytokine production was linked to increased VDAC recruitment into the mPTP. This would also indicate that there is increased HK recruitment to the mPTP as well and further limiting glycolytic ATP available in the cytosol to trigger IL-1β cleavage. Given VDAC’s role in ETC function, it is not surprising that increased mPTP activity may facilitate increased ETC activity in conditions that require mitochondrial function.

While our results demonstrate decreased inflammatory cytokine production in siTRNT1-transfected RAW264.7 cells, these findings differ from those reported by Giannelou and colleagues (15), who reported increased inflammatory cytokine levels in patient serum, patient-sourced monocyte-derived macrophages (MDMs) and transfected THP-1 cells. Several key differences may underline this discrepancy. First, SIFD can present with autoimmune features such as Type I IFN hypersignalling and protein misfolding events leading to proteasome-associated autoinflammatory syndromes (PRAAS), both of which can increase pro-inflammatory cytokines in patient serum (67). Our study design, which focused on more acute responses, may not have captured such autoinflammatory processes. Second, the authors assessed MDMs from a single patient that were differentiated using GM-CSF, which skews macrophages towards a more pro-inflammatory state (110, 111) before subsequent treatment with LPS & IFN-γ, which differs to our study. Further, their THP-1 experiments employed a longer-term siRNA protocol by electroporating the cells in their monocytic state, differentiating them with PMA for 48 hours followed by a 5-day rest period before LPS treatment. Since the authors did not report the efficiency of their siRNA knockdown, it is possible that the combination of siRNA transfection, PMA differentiation and rest period reduced the efficiency of TRNT1 knockdown, given that siRNA gene silencing typically lasts 5–7 days (112, 113). By comparison, we observed a high initial transfection efficiency in THP-1 monocytes, which dissipated 48 and 72 hours later (**Supplementary Figure 1**). As such, their longer-term approach may better represent a chronic examination of TRNT1 gene silencing on macrophage function, consistent with their comparisons to patients’ samples. Whereas our study focused on more acute effects which allow for possible mechanistic examination, with experiments lasting 1–3 days post-siRNA transfection. Cumulatively, these differences may explain the contrasting cytokine profiles observed.

While macrophage cell lines are valuable for studying macrophage responses *in vitro*, there are some limitations to their use. Firstly, the mutations that allow the cell lines to evade normal cellular senescence and proliferate are not a native characteristic to primary cells (114, 115) and may alter their cellular biology and their bioenergetics (116, 117). But their homogeneous genetic background, cost-effectiveness, and ease of manipulation make them useful for preliminary research with minimal phenotypic variability (115, 118). This study used the RAW264.7 cell line, which has historically been used as a model system to assess macrophage function and shows some translatability into the human system (119), and exhibited higher siRNA knockdown efficiency compared to other macrophage systems such as the murine BMDM and differentiated or undifferentiated human THP-1 cells under similar conditions (**Supplementary Figure 1**). While this highlights the suitability of using RAW264.7 cells for downstream applications, future work must expand into either optimized primary macrophage systems or *in vivo* mouse or patient models to assess their validity as well as their translatability to the human body.

Additionally, this study highlights the importance of mitochondria to drive proper reprogramming of macrophage function and how under disease conditions, the disturbance of this reprogramming impairs macrophage inflammatory capacity. Macrophages perform a multitude of essential functions that may also be impaired due to the absence of TRNT1. Like other phagocytic cells, such as neutrophils, macrophages clear infection by phagocytosing pathogenic threats and apoptotic cells *via* partial regulation by ICAM-1 (120–122) as well as drive the activation and proliferation of adaptive immune cells *via* antigen presentation and the secretion of IL-15 (123, 124). As such, future studies should examine the effects of TRNT1 deficiency on other macrophage functions such as phagocytosis and the facilitation of adaptive immune activation. Another limitation regards the use of PK11195 to stabilize TSPO. PK11195 is one of the most widely used TSPO ligands in research and along with 4’-cholorodiazepam (Ro5-4864), they represent the first generation of TSPO ligands (46, 125). But given the potential off-target effects (59, 99–102) and the minimal changes in function seen with PK11195 under TRNT1 deficiency, using a different TSPO ligand may provide more comparable results to TSPO overexpression. Examples include using another first-generation ligand like Ro5–4864 or newer ligands like CLINME, XBD173 or MGV-1.

**Figure 9:**
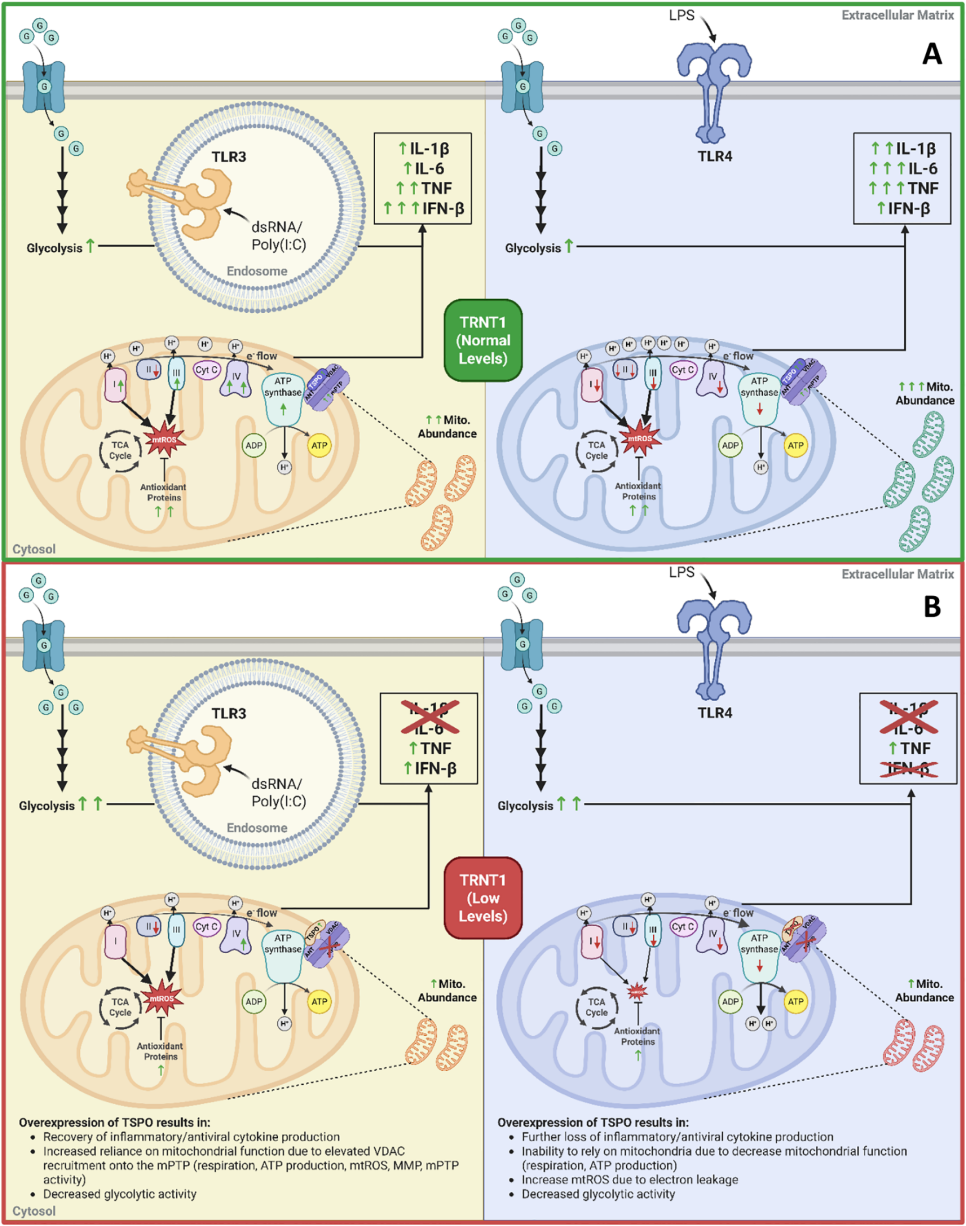
The role of low TRNT1 levels in altering macrophage metabolic reprogramming and their effector responses. Under normal conditions, LPS and Poly(I:C) activations result in distinct reprogramming of the mitochondria to elicit their respective macrophage effector responses **(A)**. Under low TRNT1 levels, this mitochondrial reprogramming, linked to the reduced/misfolded TSPO protein, is mainly lost **(B)**. Created in https://BioRender.com.

In summary, we found that TRNT1 plays a key, but distinct role in LPS and Poly (I:C)-stimulated RAW264.7 cells (**Figure 9**). Both activation states impact mitochondrial function and inflammatory cytokine expression due to the differential modulation of their respective ETC architecture (**Figure 9**). Bioinformatic analysis of mRNA transcripts enriched in codons most affected by TRNT1 loss identified TSPO as a key protein differentially regulated in the absence of TRNT1. TSPO overexpression, but not its ligand stabilization, restored the siTRNT1-mediated decrease in inflammatory cytokine production in Poly (I:C)-treated macrophages, but not LPS-treated macrophages (**Figure 9**). This was linked to increased recruitment of VDAC onto the mPTP, which resulted in elevated respiration, mPTP activity, and mtROS production. Our data suggests that mitochondrial reprogramming associated with specific responses to different pathogens could influence the ability to reverse SIFD-linked immunodeficiency, through the targeting of TSPO expression. Thus, highlighting TSPO as a potentially novel therapeutic target for SIFD-related immune pathologies.

## Data Availability

The original contributions presented in the study are included in the article/**Supplementary Material**. Further inquiries can be directed to the corresponding author.
