# Supplementary figures and images for: SIFD-associated TRNT1 deficiency unveils importance of TSPO during macrophage antibacterial and antiviral responses
Source: Front Immunol. 2025 Sep 11;16:1497766. doi: 10.3389/fimmu.2025.1497766 (PMC12460316; doi:10.3389/fimmu.2025.1497766)

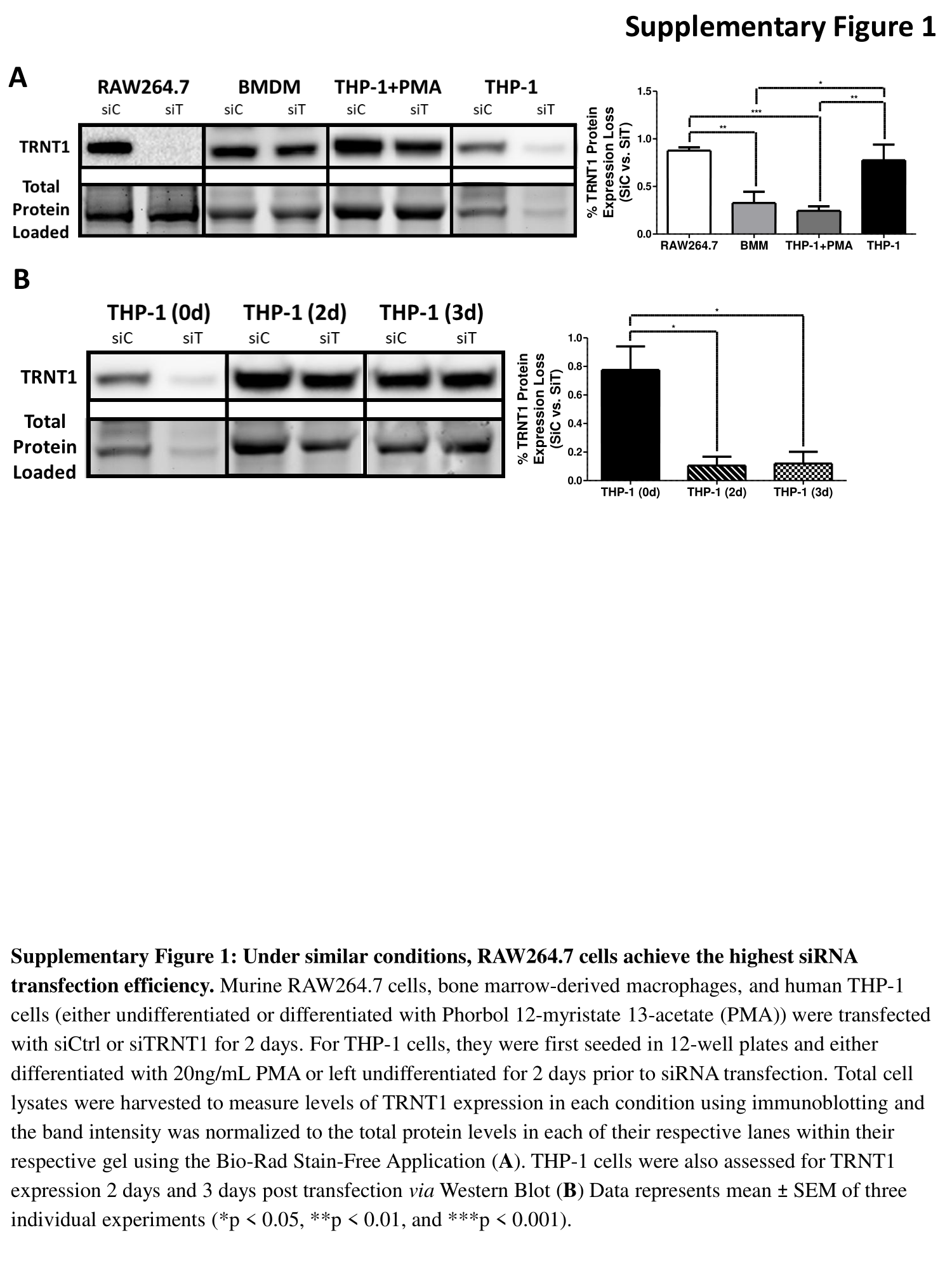

Supplement: Supplementary file 1 [file Image1.tiff]

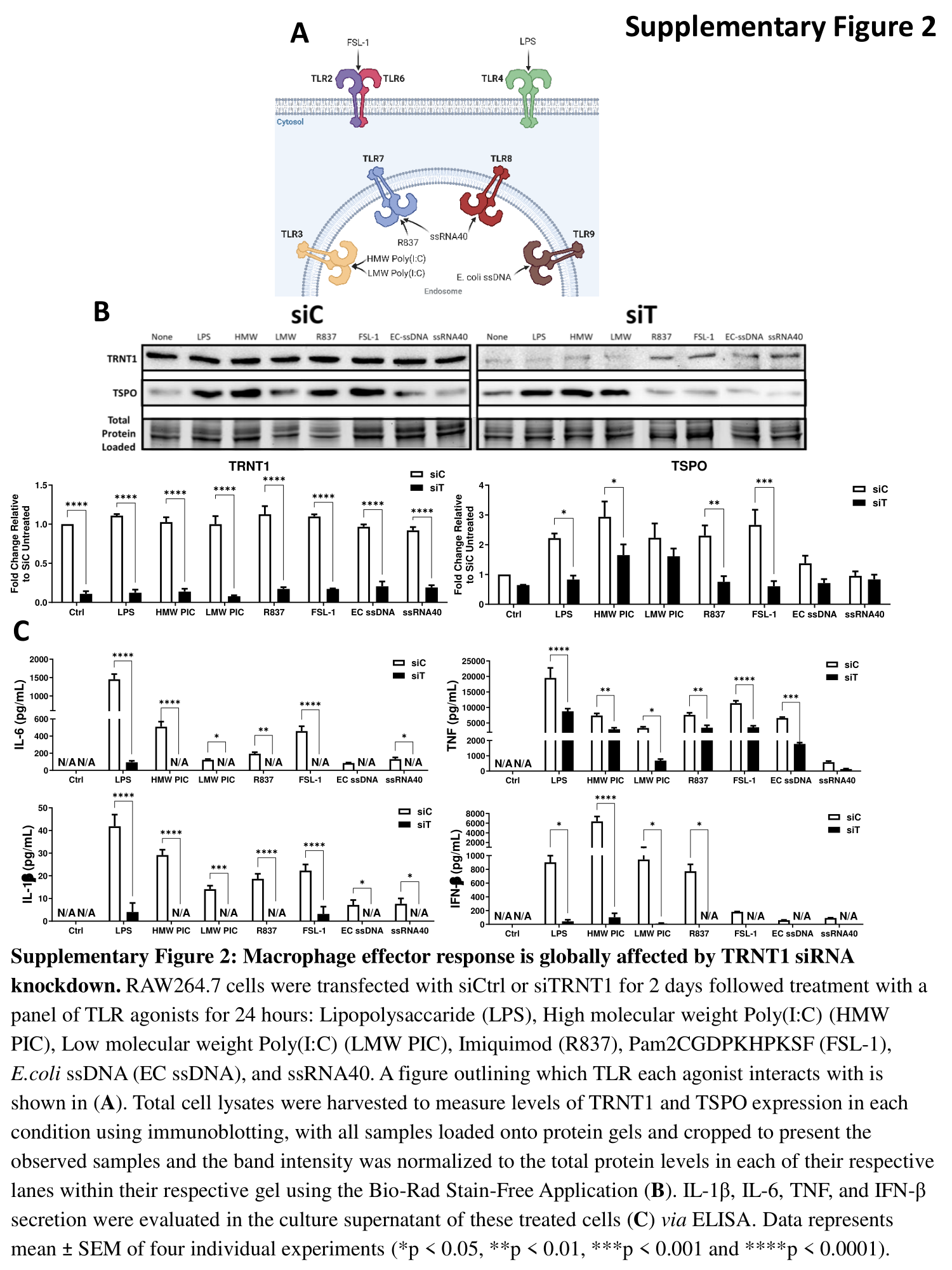

Supplement: Supplementary file 2 [file Image2.tiff]

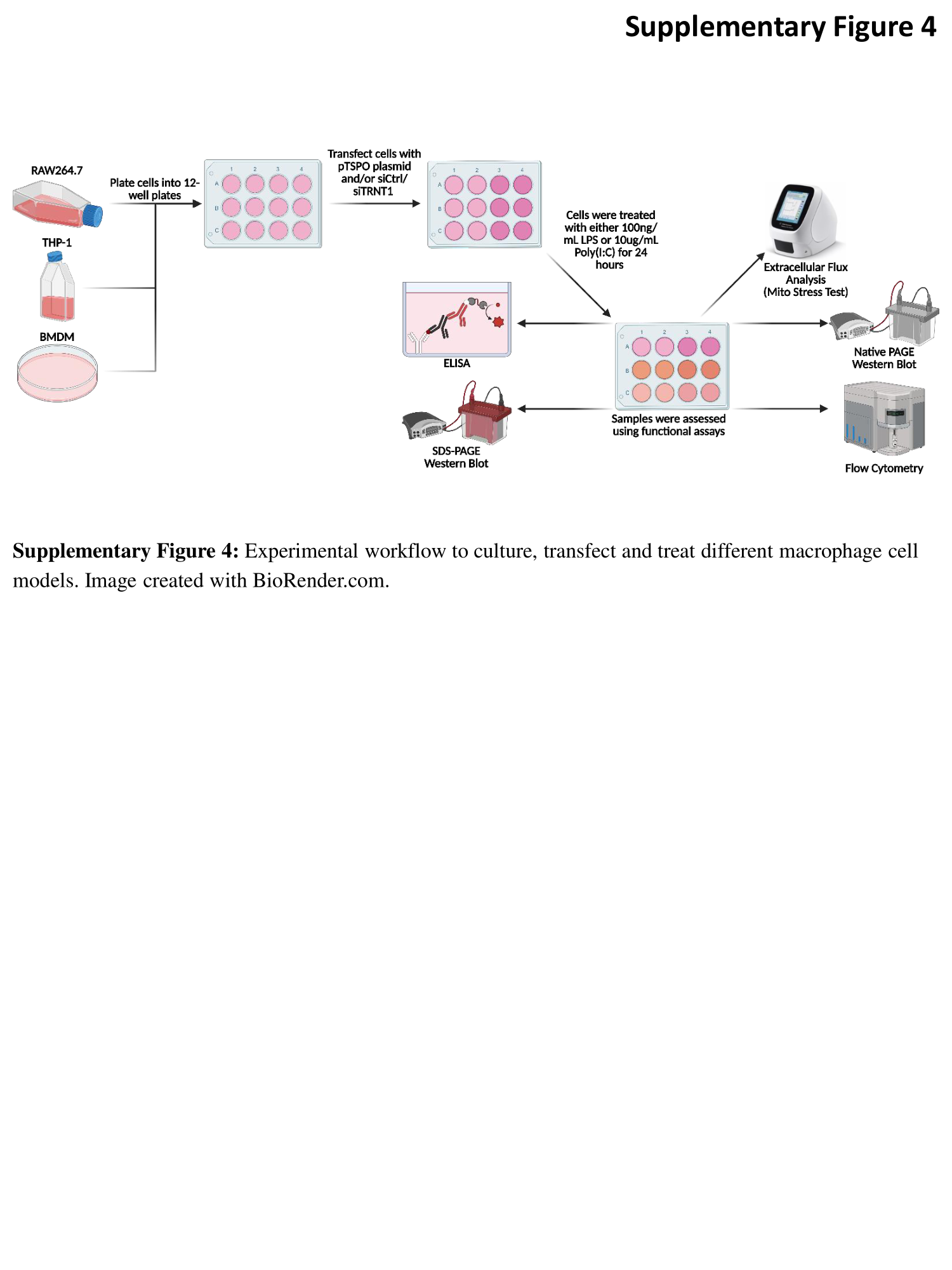

Supplement: Supplementary file 4 [file Image4.tiff]

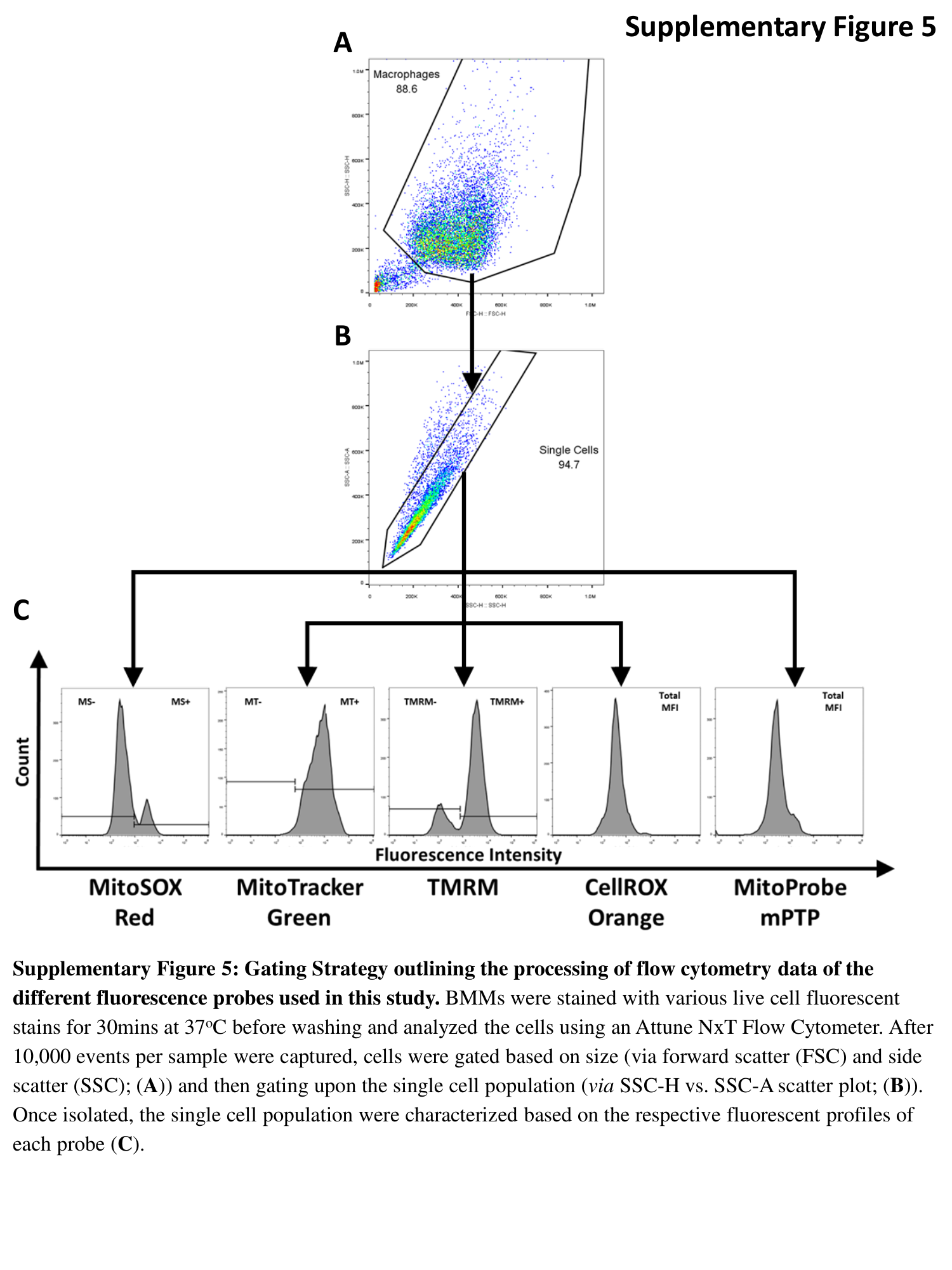

Supplement: Supplementary file 5 [file Image5.tiff]

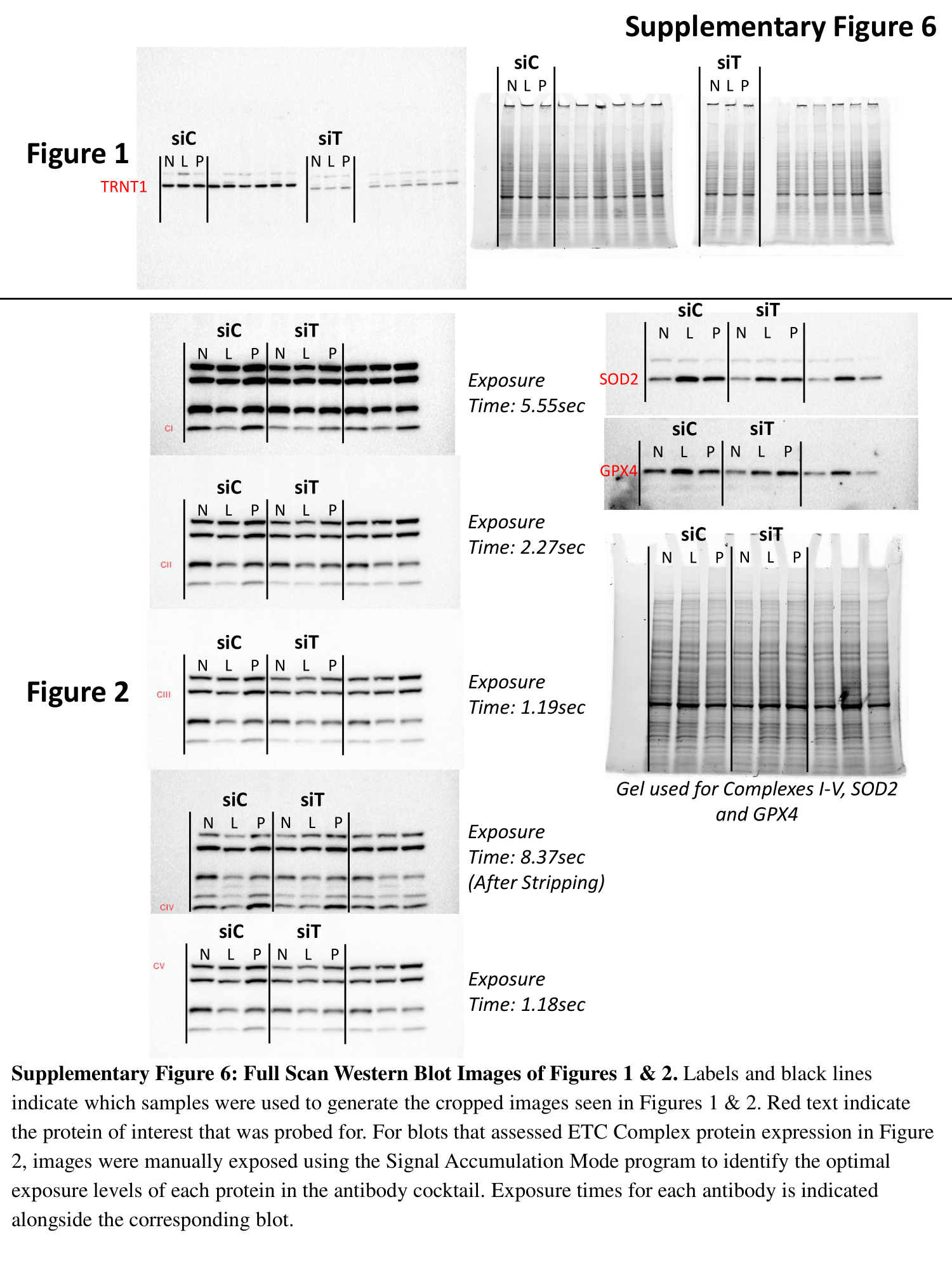

Supplement: Supplementary file 6 [file Image6.tiff]

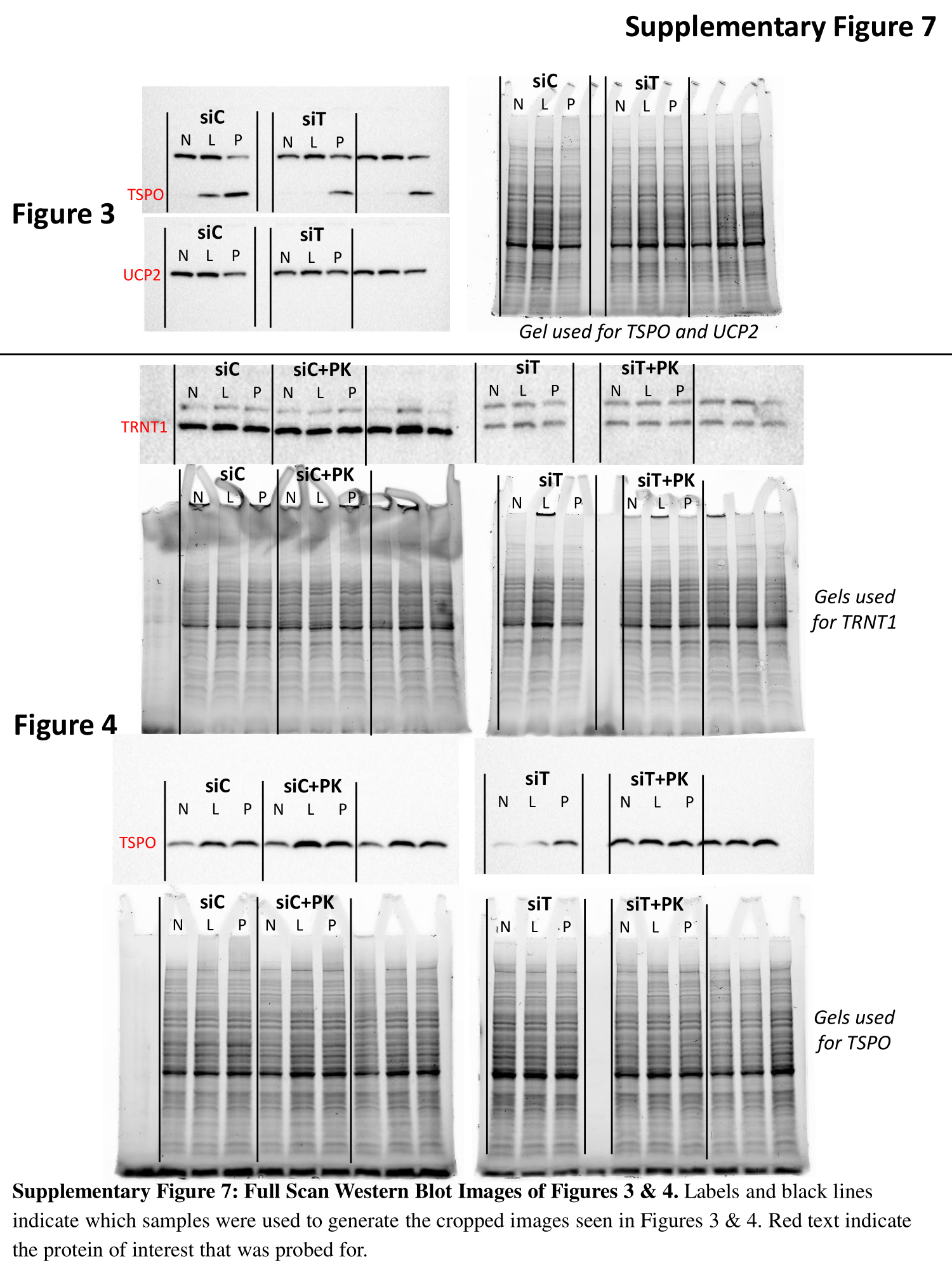

Supplement: Supplementary file 7 [file Image7.tiff]

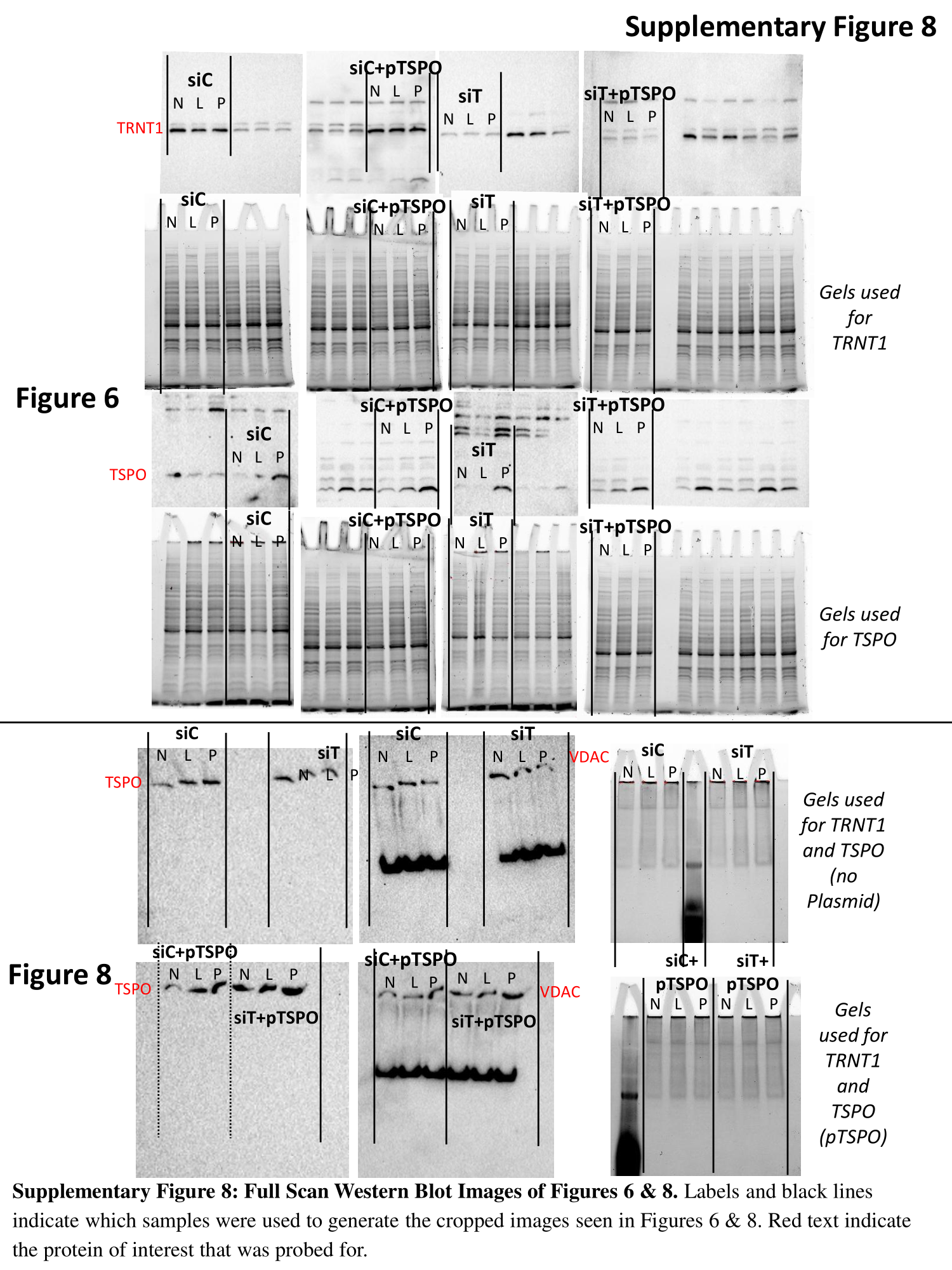

Supplement: Supplementary file 8 [file Image8.tiff]

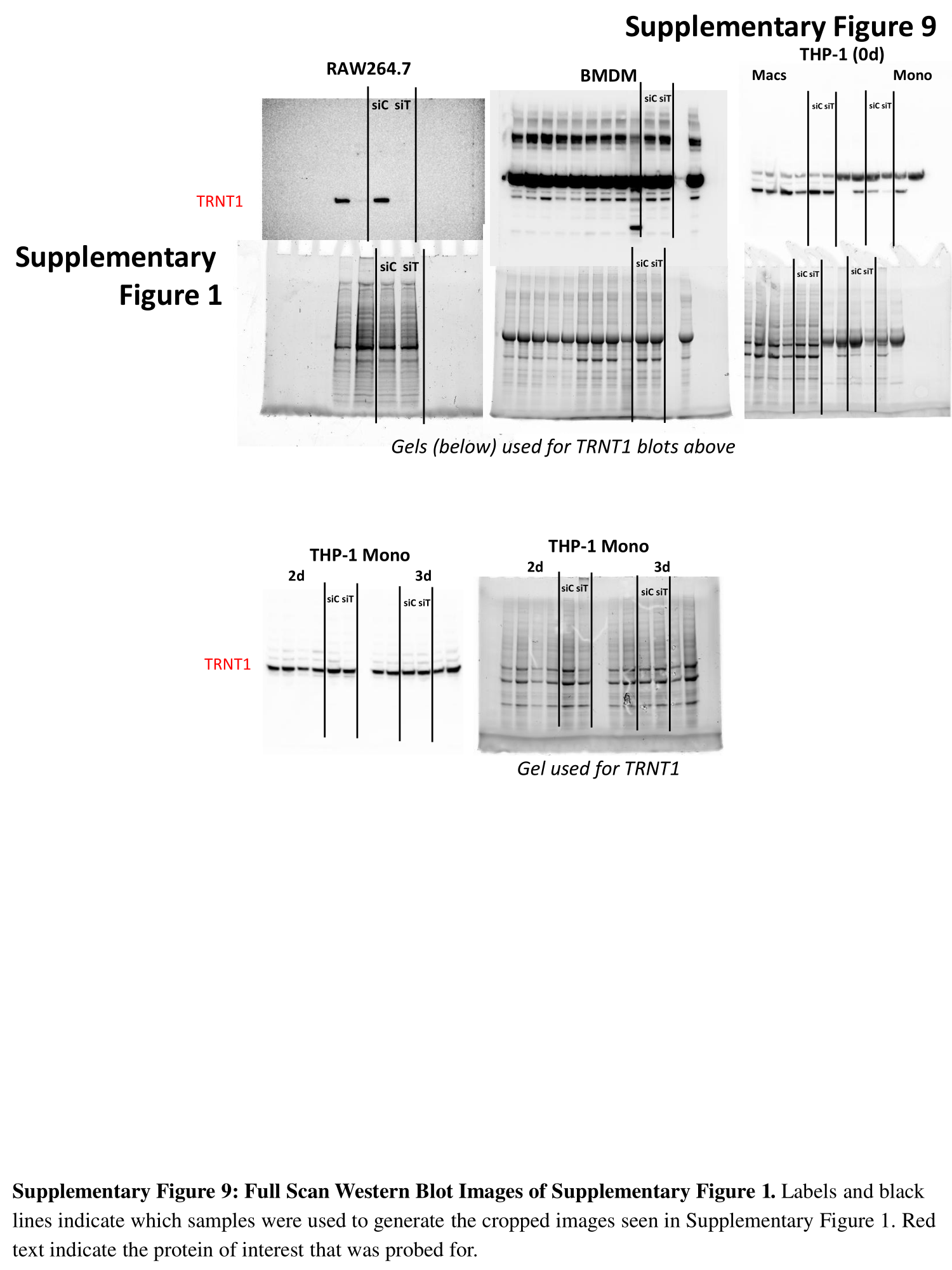

Supplement: Supplementary file 9 [file Image9.tiff]

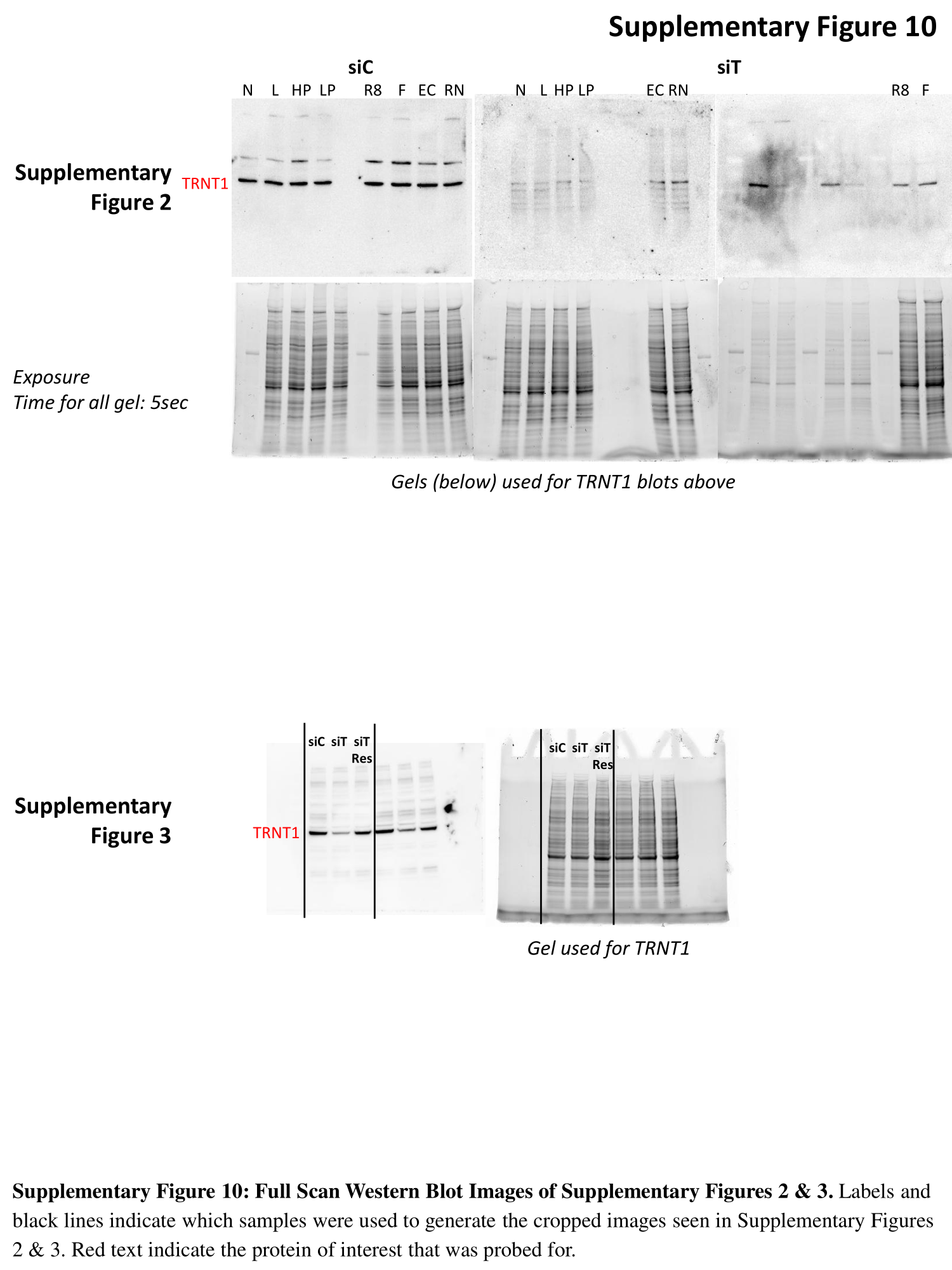

Supplement: Supplementary file 10 [file Image10.tiff]
